# Measuring and reducing the carbon footprint of fMRI preprocessing in fMRIPrep

**DOI:** 10.1002/hbm.70003

**Published:** 2024-08-26

**Authors:** Nicholas E. Souter, Nikhil Bhagwat, Chris Racey, Reese Wilkinson, Niall W. Duncan, Gabrielle Samuel, Loïc Lannelongue, Raghavendra Selvan, Charlotte L. Rae

**Affiliations:** ^1^ School of Psychology University of Sussex Brighton UK; ^2^ McConnell Brain Imaging Centre, The Neuro (Montreal Neurological Institute – Hospital) McGill University Montreal Quebec Canada; ^3^ Sussex Neuroscience University of Sussex Brighton UK; ^4^ Department of Physics and Astronomy University of Sussex Brighton UK; ^5^ Graduate Institute of Mind, Brain and Consciousness Taipei Medical University Taipei Taiwan; ^6^ Department of Global Health and Social Medicine, King's College London London UK; ^7^ Cambridge Baker Systems Genomics Initiative, Department of Public Health and Primary Care University of Cambridge Cambridge UK; ^8^ British Heart Foundation Cardiovascular Epidemiology Unit, Department of Public Health and Primary Care University of Cambridge Cambridge UK; ^9^ Victor Phillip Dahdaleh Heart and Lung Research Institute University of Cambridge Cambridge UK; ^10^ Health Data Research UK Cambridge Wellcome Genome Campus and University of Cambridge Cambridge UK; ^11^ Department of Computer Science University of Copenhagen Copenhagen Denmark; ^12^ Department of Neuroscience University of Copenhagen Copenhagen Denmark

**Keywords:** Carbon, Computing, fMRI, fMRIPrep, footprint, neuroimaging, preprocessing

## Abstract

Computationally expensive data processing in neuroimaging research places demands on energy consumption—and the resulting carbon emissions contribute to the climate crisis. We measured the carbon footprint of the functional magnetic resonance imaging (fMRI) preprocessing tool fMRIPrep, testing the effect of varying parameters on estimated carbon emissions and preprocessing performance. Performance was quantified using (a) statistical individual‐level task activation in regions of interest and (b) mean smoothness of preprocessed data. Eight variants of fMRIPrep were run with 257 participants who had completed an fMRI stop signal task (the same data also used in the original validation of fMRIPrep). Some variants led to substantial reductions in carbon emissions without sacrificing data quality: for instance, disabling FreeSurfer surface reconstruction reduced carbon emissions by 48%. We provide six recommendations for minimising emissions without compromising performance. By varying parameters and computational resources, neuroimagers can substantially reduce the carbon footprint of their preprocessing. This is one aspect of our research carbon footprint over which neuroimagers have control and agency to act upon.

## Graphical abstract

Computing needed for data processing has a carbon footprint. We estimated the carbon emissions arising from eight variants of functional magnetic resonance imaging (fMRI) preprocessing pipeline fMRIPrep. Emissions can be reduced substantially without comprising preprocessing performance, measured using statistical activation and data smoothness. For instance, disabling FreeSurfer surface reconstruction reduced average emissions by 48%.

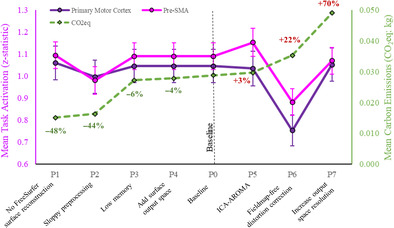

**Practitioner Points**

- Computing needed for fMRI data processing uses energy, and therefore has a carbon footprint.
- We estimated carbon emissions and pre‐processing performance for eight variants of fMRIPrep, using stop signal data for 257 subjects.
- Switching off FreeSurfer surface construction can reduce carbon emissions by up to 48% with no loss in preprocessing performance.

## 1 INTRODUCTION

We are in the midst of a climate crisis. According to the IPCC (2023), the release of greenhouse gases as a result of human activity has led to ~1.09°C warming of global surface temperatures, when comparing the period of 2011–2020 to pre‐industrial levels (1850–1900). More recent estimates from NASA suggest that average surface temperature in 2023 was—alarmingly—over 1.3°C warmer than in this same preindustrial period (https://climate.nasa.gov/vital-signs/global-temperature/). Such warming contributes to extreme weather events, including fires, droughts, and floods, and a collapse in biodiversity (IPCC, 2022). As it is not yet possible to rely on carbon‐negative technology (Carton et al., 2020) or offsetting (Watt, 2021), behaviour change is needed to combat this crisis. As scientists, we must accept the contribution of scientific practices (Valero, 2023). This can be seen in biomedical research, including in neuroscience, where carbon intensive practices include extraction of experimental resources, creation of single‐use consumables, and long‐haul flights to conferences (Aron et al., 2020; Epp et al., 2023; Farley, 2022; Keifer & Summers, 2021; Nathans & Sterling, 2016; Rae, 2022; Rae et al., 2022; Zak et al., 2020). One often neglected aspect of this research footprint is emissions associated with data processing and storage.

The information communication technology sector is responsible for an estimated 1.8%–3.9% of global CO_2_ emissions (Freitag et al., 2021)—largely attributable to electricity required to run, maintain, and cool servers (Khalaj et al., 2016). Compute‐heavy research fields include machine learning (Anthony et al., 2020), astrophysics (Portegies Zwart, 2020), and bioinformatics (Grealey et al., 2022). Tools have been developed to estimate the carbon emissions of such computing—including the online calculator Green Algorithms (Lannelongue et al., 2021), server‐side tool GA4HPC (https://www.green-algorithms.org/GA4HPC), and embedded packages Carbontracker (Anthony et al., 2020) and CodeCarbon (Goyal‐Kamal et al., 2021). Such tools estimate energy used during computing, which is used in conjunction with the carbon intensity of the local energy supply to estimate emissions. This is a useful first step in addressing the footprint of one's computing (Lannelongue et al., 2023; Lannelongue & Inouye, 2023).

Neuroimaging research frequently relies on computationally expensive processing with large datasets (Rae et al., 2022). This includes statistical analysis of brain structure and function, and preprocessing needed to prepare data for analysis. For functional magnetic resonance imaging (fMRI), preprocessing steps include registration into a standard template space, extraction (identifying brain tissue), segmentation between tissue type boundaries, distortion correction, and identification of nuisance regressors (Caballero‐Gaudes & Reynolds, 2017). fMRIPrep is a preprocessing pipeline which combines best practice tools from multiple packages (Esteban et al., 2019, 2020). fMRIPrep runs several options by default, including surface reconstruction in FreeSurfer and registration and extraction in Advanced Normalization Tools (ANTs). Its command line interface provides the option to add, remove, or adjust preprocessing steps—which in turn impact runtime, files generated, and computational resources needed. As of version 22.1.0 (12 December 2022), CodeCarbon has been integrated into fMRIPrep (https://fmriprep.org/en/stable/changes.html#december-12-2022), allowing users to estimate carbon emissions for a subject's preprocessing. fMRIPrep is therefore well placed to test the tractability of reducing the carbon footprint of a neuroimaging pipeline.

Here, we measured the impact of preprocessing parameters in fMRIPrep on carbon emissions across eight pipeline variants. We also tracked preprocessing performance using two metrics used in the original validation of fMRIPrep (Esteban et al., 2019)—statistical task activation and mean data smoothness. In estimating both carbon emissions and preprocessing performance, we aimed to identify settings that minimise carbon emissions while providing little or no trade‐off in the quality of derived image phenotypes. While insufficient data exists to formulate specific hypotheses, adding preprocessing steps (e.g., distortion correction) should increase carbon emissions while benefitting pipeline performance. Conversely, making pipelines more computationally efficient (e.g., reducing memory usage) should decrease emissions while having no impact on preprocessing quality. Beyond this, analysis was exploratory.

The results reported in this article have been reproduced by an independent statistician from the University of Sussex (report: https://osf.io/hzu69).

## 2 METHODS

### 2.1 Preregistration

This project was preregistered on the OSF (https://osf.io/839pa) on 31 March 2023. This preregistration was created using the Psychological Research Preregistration‐Quantitative (PRP‐QUANT) Template, version 2 (available at https://www.psycharchives.org). Several deviations were made from this preregistration plan. Each is detailed in the Supplementary Section ‘Deviations from preregistration’.

### 2.2 Participants

We used existing data from the publicly available UCLA Consortium for Neuropsychiatric Phenomics LA5c Study (Bilder et al., 2020; Gorgolewski et al., 2017; https://openneuro.org/datasets/ds000030/versions/1.0.0). A ‘data descriptor’ of this project is provided by Poldrack et al. (2016). This dataset includes 272 participants including those with a diagnosis of ADHD (*N* = 43), bipolar disorder (*N* = 29), or schizophrenia (*N* = 50) as well as neurologically healthy controls (*N* = 130). Participants completed a series of MRI scans including structural scans, diffusion‐weighted imaging, resting state fMRI, and task‐based fMRI for six tasks. We analysed functional data for the stop signal task only. Data for this same sample and task were used in the original validation of fMRIPrep (Esteban et al., 2019) when comparing fMRIPrep and FSL FEAT. Participants were necessarily excluded if they did not have data for a T1 structural scan or the single run of the stop signal task (*N* = 13). Consistent with Esteban et al. (2019), we also excluded participants who had no valid trials classified as successful go (*N* = 1) or successful stop (*N* = 1). The final sample included 257 participants (108 female) with a mean age of 33.3 (SD = 9.3).

### 2.3 Design

This study had a repeated measures design, with raw fMRI data for all 257 participants being run through eight fMRIPrep pipeline variants.

### 2.4 Procedure

#### 2.4.1 Stop signal task

The procedure used for the stop signal task is described fully in Poldrack et al. (2016). Participants completed one run of this task during fMRI. Each participant completed 128 trials; 96 ‘go’ and 32 ‘stop’. Each trial was preceded by a 500 ms fixation cross. Participants were then presented with an arrow pointing left or right, indicating the response they should provide, which remained visible for 1000 ms (the decision period). Using a button box in their right hand, participants were instructed to indicate the direction of the stimulus arrow on ‘go’ trials. Participants were instructed to inhibit these button presses during ‘stop’ trials, on which they heard a 500 Hz audio tone. The delay period between the arrow appearing and the auditory ‘stop’ stimulus for a given trial increased by 50 ms intervals after previous successful inhibition of a motor response and decreased by 50 ms intervals following failed inhibitions. Audio stop signals lasted for 250 ms. Trials were separated by jittered null periods during which a blank screen was presented. Null periods ranged from 500 to 4000 ms, with an average of 1000 ms.

#### 2.4.2 Preprocessing in fMRIPrep

A baseline fMRIPrep pipeline was run on the stop signal task data for all 257 subjects. Flags passed to this pipeline are in Table 1. We ran seven variants on this pipeline for all subjects. Table 2 presents a numeric ‘ID’, descriptive ‘label’, and description for each variant. Largely default settings were used for the baseline pipeline, with the exception of using ‘MNI152NLin6Asym:res‐2’ as an output space, rather than the default of ‘MNI152NLin2009cAsym’ in native resolution. Doing so allowed for a fairer comparison between the baseline pipeline and the pipeline employing the independent component analysis strategy for the Automatic Removal of Motion Artifacts (ICA‐AROMA; P5). Two further pipelines manipulating parallelisation of computing are described and analysed in the Supplementary Section ‘Parallelisation analysis’ (Tables S1–S3 and Figures S1 and S2), including a discussion of why they were not included in the main analysis (Figure s3).

**TABLE 1:** Flags passed to the baseline fMRIPrep pipeline.

Flag	Description
‐‐ignore **slicetiming**	Tells fMRIPrep not to perform slicetiming correction—this step was not deemed necessary for this study.
‐‐track‐carbon	Runs CodeCarbon during preprocessing, providing a subject‐specific estimate of total carbon emissions, duration, and energy consumption.
‐‐country‐code **GBR**	Specifies the country in which CodeCarbon has been run using the relevant ISO Alpha‐3 country code, in this case for Great Britain.
‐‐random‐seed **1234**	Initiates a specific seed for the workflow. Along with the two following flags below, this helps to ensure reproducible preprocessing.^a^
‐‐skull‐strip‐fixed‐seed	Avoids using a random seed for skull‐stripping. Again, this ensures reproducible preprocessing when used with the flag above and below.
‐‐omp‐nthreads **1**	The maximum number of threads allocated per process. Setting this at a value of 1 ensures reproducible preprocessing when used with the two flags above.
‐‐nthreads **5**	The number of threads used across all processes, set at a default value of 5.
‐‐mem‐mb **3000**	Sets an upper bound memory limit for fMRIPrep processes, at 3000 MB.
‐‐output‐spaces **MNI152NLin6Asym:res‐2**	Specifies the output space in which to register fMRI data. Outputs data to a template with standard 2 mm resolution.
‐‐skip‐bids‐validation	This flag uses the assumption that input data is BIDS‐valid (this was verified using the BIDS Validator prior to preprocessing; https://bids‐standard.github.io/bids‐validator/). When used, BIDS validation is skipped within fMRIPrep.

*Note*: Excludes arguments used to identify the location of relevant directories and licences. Values or strings given to arguments (when relevant) are in bold.

^a^ See https://mattermost.brainhack.org/brainhack/channels/fmriprep_reproducibility for a discussion on reproducibility within fMRIPrep.

**TABLE 2:** Description of fMRIPrep pipeline variants.

ID	Label	Flag addition	Description and predicted impact
0	Baseline	N/A	The baseline fMRIPrep command with Table 1 flags only. All predicted directions of effects below are relative to this baseline.
1	No FreeSurfer surface reconstruction	‐‐fs‐no‐reconall	Removes the default FreeSurfer brain surface reconstruction. This can be expected to decrease carbon emissions while not affecting preprocessing performance, as surface reconstruction should not impact volumetric preprocessing.
2	Sloppy preprocessing	‐‐sloppy	Implements the ‘sloppy’ preprocessing mode intended for testing pipelines, which saves time by performing low quality registration. This can be expected to reduce carbon emissions while markedly decreasing preprocessing performance (by design).
3	Low memory	‐‐low‐mem	Attempts to reduce the memory usage of fMRIPrep, while increasing disk usage in the working directory. By restricting the resources needed for preprocessing, this may reduce carbon emissions while not impacting preprocessing performance.
4	Add surface output space	‐‐output‐spaces **MNI152NLin6Asym:res‐2 fsaverage**	Adds a surface output space alongside the volumetric space. This is predicted to increase carbon emissions while having no effect on preprocessing performance (given that data resampled to this surface space was not used in subsequent analysis steps that analysed data in volumetric space only).
5	ICA‐AROMA	‐‐use‐aroma	Adds an independent components analysis (ICA) approach to automatic removal of motion artifacts (AROMA) to the pipeline. Predicted to increase carbon emissions, while improving statistical sensitivity to task activation.
6	Fieldmap‐free distortion correction	‐‐use‐syn‐sdc **error**	fMRIPrep's in‐house fieldmap‐free distortion correction, using the subject's T1w image as an anatomically unwarped reference. Predicted to increase emissions. This should theoretically improve preprocessing performance.
7	Increase output space resolution	‐‐output‐spaces **MNI152NLin6Asym:res‐1**	Increases the resolution of the volumetric output space used to resample anatomical and functional images, from 2 mm to 1 mm. Predicted to increase both carbon emissions and preprocessing performance.

*Note*: ‘flag addition’ refers to the respective arguments passed to the fMRIPrep command line (https://fmriprep.org/en/stable/usage.html). Values or strings given to arguments (when relevant) are in bold.

Further details on the steps implemented for each pipeline can be seen in the Supplementary Section ‘fMRIPrep citation boilerplates’. All variants of fMRIPrep were run on the University of Sussex high performance cluster, comprising 1250 central processing unit (CPU) cores across ~90 nodes. fMRIPrep was run in a singularity container, using version 22.1.1. All jobs were run on nodes using an Intel® Xeon® Processor E5‐2640 v3.^1^

For preprocessing in fMRIPrep for each pipeline, we measured the size (GB) of files generated for each subject.

#### 2.4.3 Carbon tracking

We had planned to estimate carbon emissions of each pipeline using CodeCarbon, which has been integrated into the fMRIPrep command line as of version 22.1.0 (https://fmriprep.org/en/stable/changes.html#december-12-2022). We encountered issues specific to our high‐performance computing (HPC) architecture, whereby CodeCarbon was measuring CPU energy used by the entire node occupied by a task including baseload, rather than energy attributable only to the task itself. This led to systematic increases or decreases in emission estimates over time that were influenced by other users' jobs. More detail is provided on this issue in the Supplementary Section ‘Complications using CodeCarbon’ (Figure S4). In short, CodeCarbon is likely to be of most use to users who are able to run fMRIPrep jobs with hardware isolation and exclusive use of a node. For our purposes, this was not possible.

Instead, we developed an in‐house tracking system that relies on retrospective use of HPC scheduler logs. Energy usage (kWh) for CPU was estimated by multiplying total CPU time by the power usage of a CPU core. The power usage of a CPU core was obtained by dividing the thermal design power (TDP; 90 W) of the node in use by the total number of available CPU cores (16). Total CPU time was obtained from the task logs and represents the sum of the active time of each CPU core used. Energy usage for random‐access memory (RAM; kWh) was estimated by multiplying maximum memory used (rounded up to the nearest GB) by volatile memory consumption (0.3725; value taken from GA4HPC) and runtime. For ~10% of subjects for each pipeline, an HPC glitch led to reporting of max memory usage of 1 TB, rather than the typical value for a given pipeline (e.g., ~9 GB). For these cases, max memory values were replaced with the mean of values from subjects for whom this glitch had not occurred, prior to energy usage calculation. Estimated emissions (gCO_2_/kWh) were calculated by multiplying the estimated total energy usage (CPU + RAM) by 193.38, the average carbon intensity value for the United Kingdom provided in the 2022 v1.0 release of Country Specific Electricity Grid Greenhouse Gas Emission Factors (2022) from www.carbonfootprint.com, and by 1.28, the estimated power usage effectiveness of the Sussex HPC architecture as determined by data centre baseline power readings measure on 19 October 2023.^2^ This approach is analogous to that used by the Green Algorithms server‐side tool GA4HPC (https://www.green-algorithms.org/GA4HPC). We extracted estimated emissions (kgCO_2_eq), duration of preprocessing (hours), and energy used by CPU and RAM (kWh).

It is important to note that there is currently no gold standard approach for estimating energy usage, and other approaches are available. For instance, one could attempt to define energy usage based on the amount of memory actually used in computing. However, total memory available/reserved for a given job may provide a more accurate measure (Karyakin & Salem, 2017), and this is the approach used in Green Algorithms (Lannelongue et al., 2021). We instead used the maximum memory employed in order to fairly account for how much memory a given task should have used, rather than merely the amount allocated (consistent across pipelines and subjects), given that on our architecture, memory used occasionally exceeded memory requested. In the current experiment, RAM energy usage for a given job accounted for an average of 28% of total energy usage (CPU + RAM; for P0), broadly consistent with prior evidence (Fukazawa et al., 2018).

#### 2.4.4 Smoothing and mean smoothness estimation in AFNI

Following preprocessing in fMRIPrep, we followed the procedure for smoothing and estimating smoothness used by Esteban et al. (2019). The preprocessed blood oxygen level dependent (BOLD) data for the stop signal task was smoothed using the AFNI tool 3dBlurInMask, with a full‐width at half‐maximum (FWHM) smoothing kernel of 5 mm. Smoothing was masked by the respective subject's brain mask provided by fMRIPrep. Mean geometric smoothness estimates were extracted for each participant's BOLD run both prior to and following smoothing, using AFNI's 3dFWHMx. Outlier voxels were accounted for using the ‘detrend’ flag. This tool provides a mean smoothness estimate for each of the 184 volumes of the BOLD run on the *x*, *y*, and *z* dimension. For each dimension, outlier smoothness estimates ± three standard deviations (SDs) from the dimension mean were removed. Values were then averaged within and across dimensions to produce a single mean smoothness estimate, for both the pre‐ and post‐smoothed data. As in Esteban et al. (2019), larger mean smoothness values were taken as reflecting poorer pipeline performance, on the basis that increased smoothness reflects loss of anatomical specificity and statistical power. Smoothing was conducted for all pipelines with the exception of Pipeline 5, given that smoothing was performed during preprocessing as part of the ICA‐AROMA procedure, with a smoothing kernel of 6 mm. As such, we were unable to obtain estimates of pre‐smoothed smoothness for this pipeline.

#### 2.4.5 Analysis in FSL

To statistically measure task‐related activation during performance of the stop signal task, we employed general linear modelling using the FEAT function of FSL (Woolrich et al., 2001). In first‐level analysis, preprocessing steps were skipped, with the exception of high‐pass temporal filtering at 100 Hz. The respective preprocessed BOLD data for the stop signal run was used as the input file for each participant. In line with the design used by Gorgolewski et al. (2017) and Esteban et al. (2019), six regressors were used as EVs:

1. Go—Trials in the ‘go’ condition for which participants responded correctly, with a fixed duration of 1.5 s.
2. Go RT—Trials in the ‘go’ condition for which participants responded correctly. Duration was set as the subject's response time (RT) for the respective trial, rather than being fixed. This EV was orthogonalised to ‘go’.
3. Successful stop—Trials in the ‘stop’ condition for which participants successfully inhibited their motor response, with a fixed duration of 1.5 s.
4. Unsuccessful stop—Trials in the ‘stop’ condition for which participants did not successfully inhibit their motor response, with a fixed duration of 1.5 s.
5. Unsuccessful stop RT—Trials in the ‘stop’ condition for which participants did not successfully inhibit their motor response. Duration was set as the subject's RT for the respective trial, rather than being fixed. This EV was orthogonalised to ‘stop_unsuccess’.
6. Erroneous—Trials in the ‘go’ condition for which participants responded incorrectly, with a fixed duration of 1.5 s. Ninety‐nine participants had no erroneous ‘go’ trials. In these cases, task data were modelled with five EVs, with ‘Erroneous’ omitted.

The onset time of each regressor was set as the start of the 0.5 s fixation that preceded each decision period.^3^ Time periods not covered by the EVs include null periods between trials, and ‘go’ trials on which participants provided no response (only 79 participants had any instance of this). As in Esteban et al. (2019), we extracted the contrast of ‘go > successful stop’, producing a statistical image in which positive values reflected voxels activated by motor response, and negative values reflected voxels activated by successful response inhibition. The reverse of this contrast was also extracted to provide ‘successful stop > go’. First‐level results were cluster‐corrected at a threshold of *Z* = 3.1 (*p* = .05).

**FIGURE 1:**
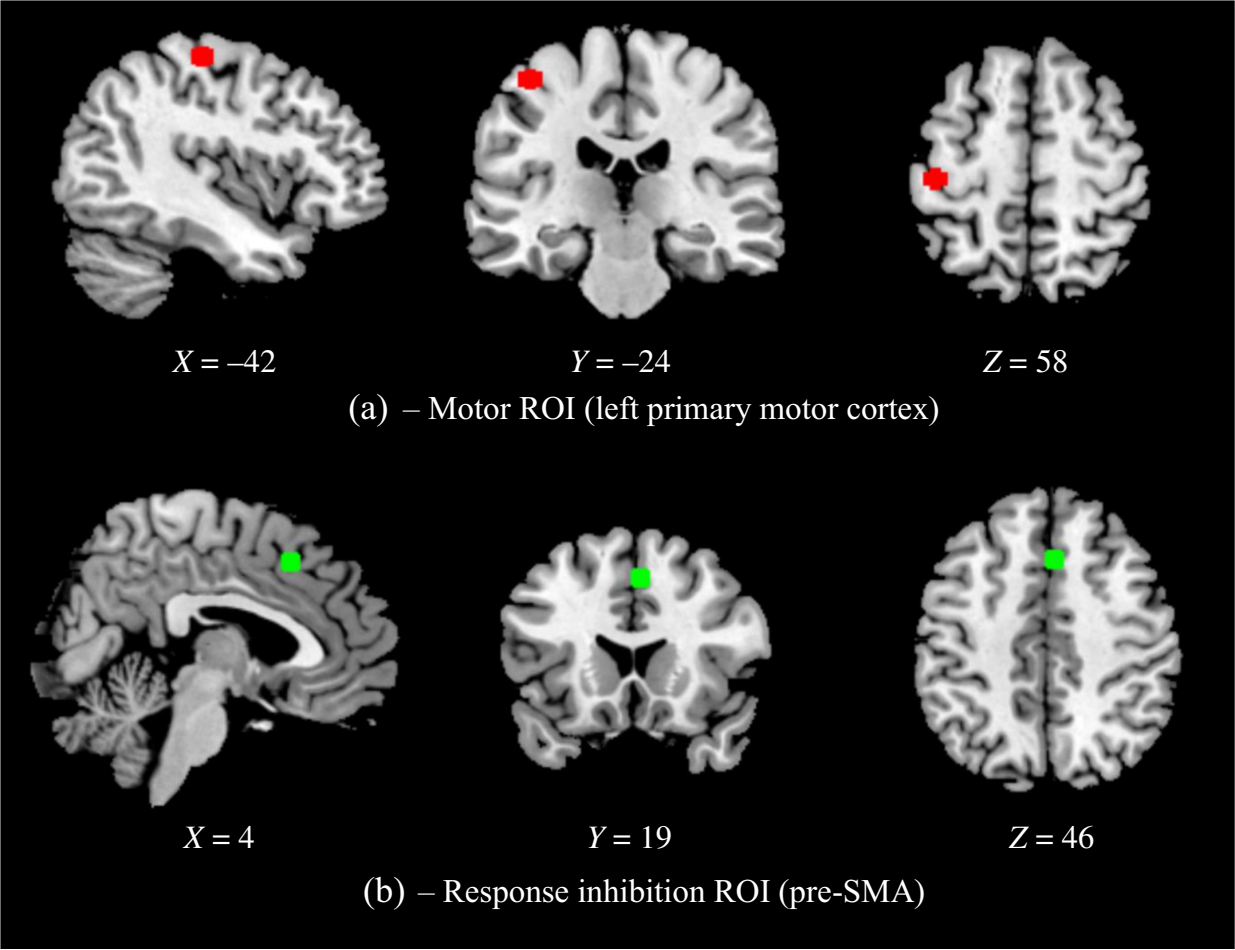
ROIs for use in FSL Featquery, including the left primary motor cortex for motor response activation, and the pre‐SMA for successful response inhibition activation. pre‐SMA, pre‐supplementary motor area; ROI, region of interest.

Following first‐level analysis, activation in two regions of interest (ROIs) was interrogated using FSL Featquery. ROIs were selected on the basis of peak activations in group‐level activation maps from analysis of this dataset by Gorgolewski et al. (2017), available on Neurovault (http://neurovault.org/collections/2606). An ROI in the left primary motor cortex (postcentral gyrus) was selected for the ‘motor’ contrast (go > successful stop). An ROI in the pre‐supplementary motor area (pre‐SMA) was selected for the ‘response inhibition’ contrast (successful stop > go). This region was selected due to its relevance to response inhibition (Rae et al., 2015; Sebastian et al., 2013; Sharp et al., 2010). In both cases, we identified the peak voxel in the respective region in the Gorgolewski et al. (2017) group map and generated a 5 mm sphere around its voxel coordinates. These ROIs are visualised in Figure 1. We extracted the mean *z*‐statistic value of voxels within each sphere for the relevant contrast (‘go’ for primary motor cortex, ‘successful stop’ for pre‐SMA). Higher mean z‐statistics in these regions, as a proxy for sensitivity to statistical activation (Esteban et al., 2019), were taken to reflect better performance.

We also performed supplementary analysis on two other ROIs relevant to the response inhibition. This included right insula (inferior opercular regions such as the insula have also been evidenced to support response inhibition; Cunillera et al., 2016; Zhang et al., 2017) and the left primary auditory cortex (due to the presentation of an auditory tone in ‘stop’ trials). Visualisation and analysis of these regions can be seen in Supplementary Section ‘Supplementary task activation’ (Figures S5 and S6 and Table S4).

Finally, higher‐level group analysis was conducted in FSL FEAT (Woolrich et al., 2004), testing for significant activation associated with ‘go > successful stop’ and ‘successful stop > go’ across the 257 subjects for each pipeline. A cluster corrected threshold of *Z* = 3.1, *p* = .05 was used.

**FIGURE 2:**
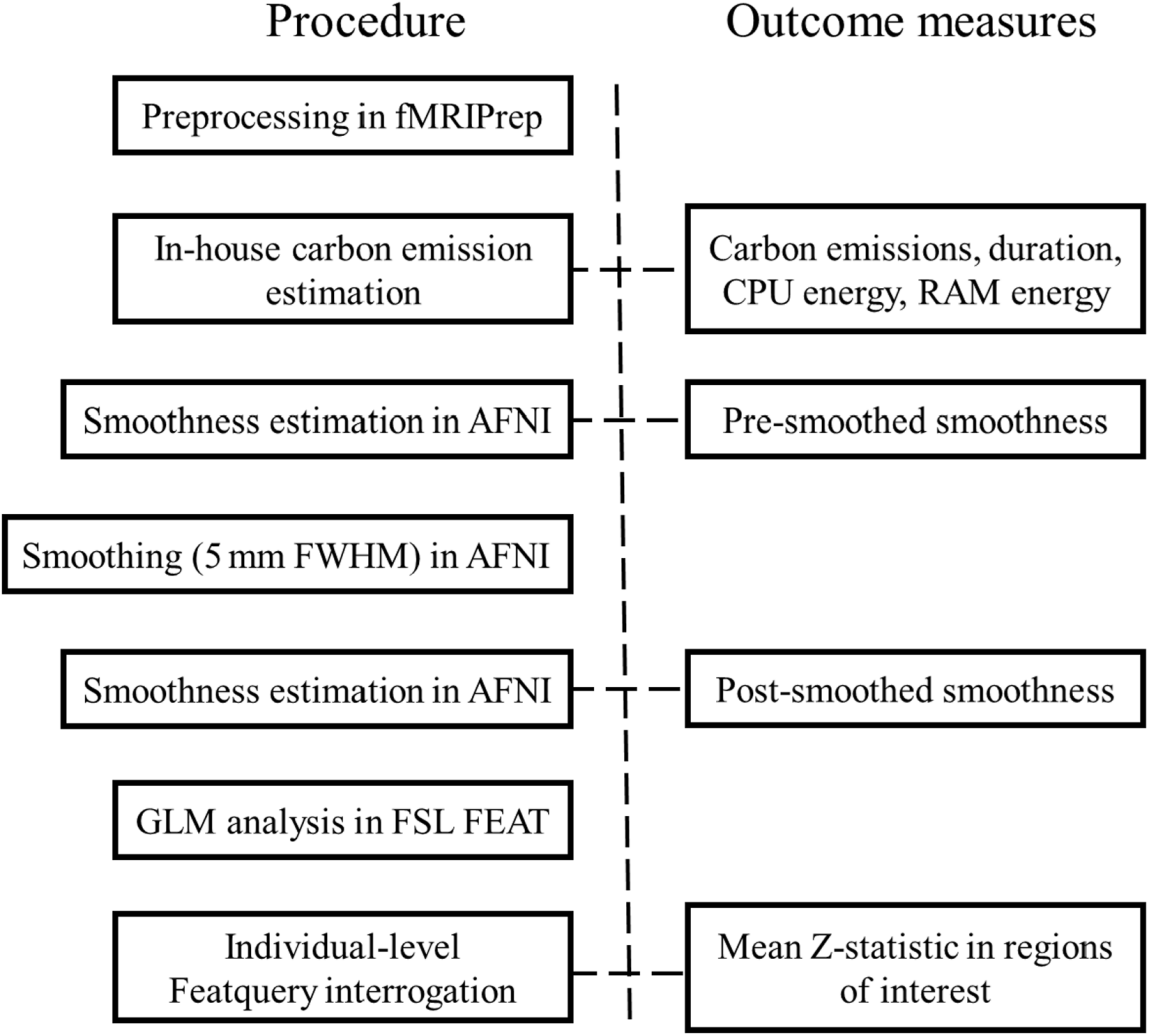
Experimental procedure of preprocessing and analysis steps, with outcome measures derived from each stage. CPU, central processing unit; GLM, generalised linear model; RAM, random‐access memory.

A summary of the experimental procedure is provided in Figure 2, alongside the output measures extracted at each stage.

### 2.5 Data analysis

To summarise, dependent variables include the carbon tracking metrics (1) carbon emissions (kgCO_2_eq), (2) duration (hours), (3) CPU energy (kWh), and (4) RAM energy (kWh), mean smoothness estimates for (5) pre‐smoothed and (6) post‐smoothed data, task activation in (7) the left primary motor cortex for motor responses and (8) the pre‐SMA for successful response inhibition, and (9) total file size. Prior to analysis, outliers (3 SD above or below the group mean) for the respective variable for the respective pipeline were removed.

For each dependent variable, we then ran a repeated measures ANOVA observing the main effect of pipeline identity, with eight levels corresponding to the pipelines detailed in Table 2 (the ANOVA for pre‐smoothed data had seven levels given that this metric was not available for P5). When significant effects of pipeline were observed, ANOVAs were followed by *t*‐test contrasts comparing each experimental pipeline (P1–P7) to the baseline pipeline (P0), with false discovery rate correction using the Benjamini–Hochberg method applied.

Note that for ANOVAs observing performance metrics (smoothness and task activation), it was not possible to perform sphericity tests or apply sphericity correction given that multiple pipelines (P0, P3, P4) presented with identical values, providing singular sum‐of‐squares‐and‐products (SSP) matrices. While this may lessen confidence in these ANOVAs, post hoc contrasts should still provide an accurate picture of pipeline differences.

Each frequentist ANOVA was followed by an equivalent repeated measures Bayesian ANOVA. For each dependent variable, this allowed us to quantify how much more likely the effect of pipeline is to be supported than the null hypothesis (no effect of pipeline). Each ANOVA was constructed using the default coefficient fixed effect prior value (0.5) with a Bayes factor of BF_10_. Each model was compared with the null model. Inference for the interpretation of observed factors was based on the classifications provided by Andraszewicz et al. (2015), originally from Jeffreys (1961). All ANOVAs and contrasts were run in the statistical package JASP version 0.17.2.1 (JASP Team, 2023).

The computational reproducibility of the results reported in this study has been checked by an independent statistician (see https://osf.io/hzu69 for the full report). This was conducted as part of the University of Sussex School of Psychology initiative ‘Ensuring the computational reproducibility of to‐be‐submitted psychology papers’ (https://doi.org/10.17605/osf.io/dr35v).

Analysis was followed by creation of activation count maps and SD maps for each pipeline, as in Esteban et al. (2019). This allowed for qualitative assessment of the anatomical specificity and variability of each pipeline. The procedure used to generate each of these maps is described in Section 3.2.

## 3 RESULTS

### 3.1 Pipeline comparisons

**FIGURE 3:**
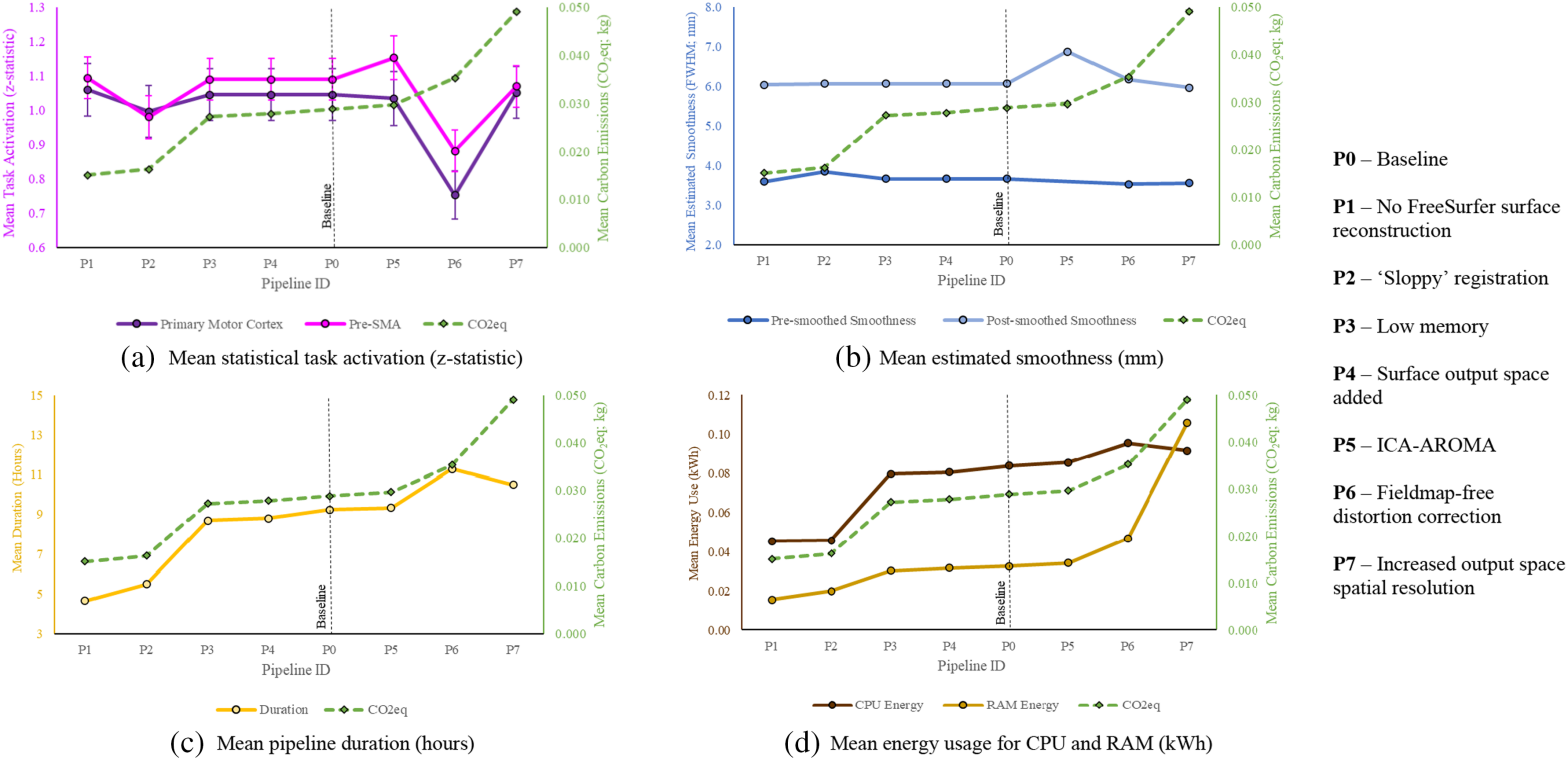
Estimated carbon emissions (dotted green line) plotted against (a) statistical task activation in regions of interest, (b) estimated data smoothness, (c) duration of preprocessing, and (d) CPU and RAM energy usage, for each pipeline. Error bars reflect one standard error of the mean. These are frequently too small to be visible. Note that the scale used varies between variables, see text below for percent changes. *N* = 257. CO_2_eq, carbon dioxide equivalent; CPU, central processing unit; kg, kilograms; kWh, kilowatt hours; mm, millimetres; pre‐SMA, pre‐supplementary motor area; RAM, random‐access memory.

**FIGURE 4:**
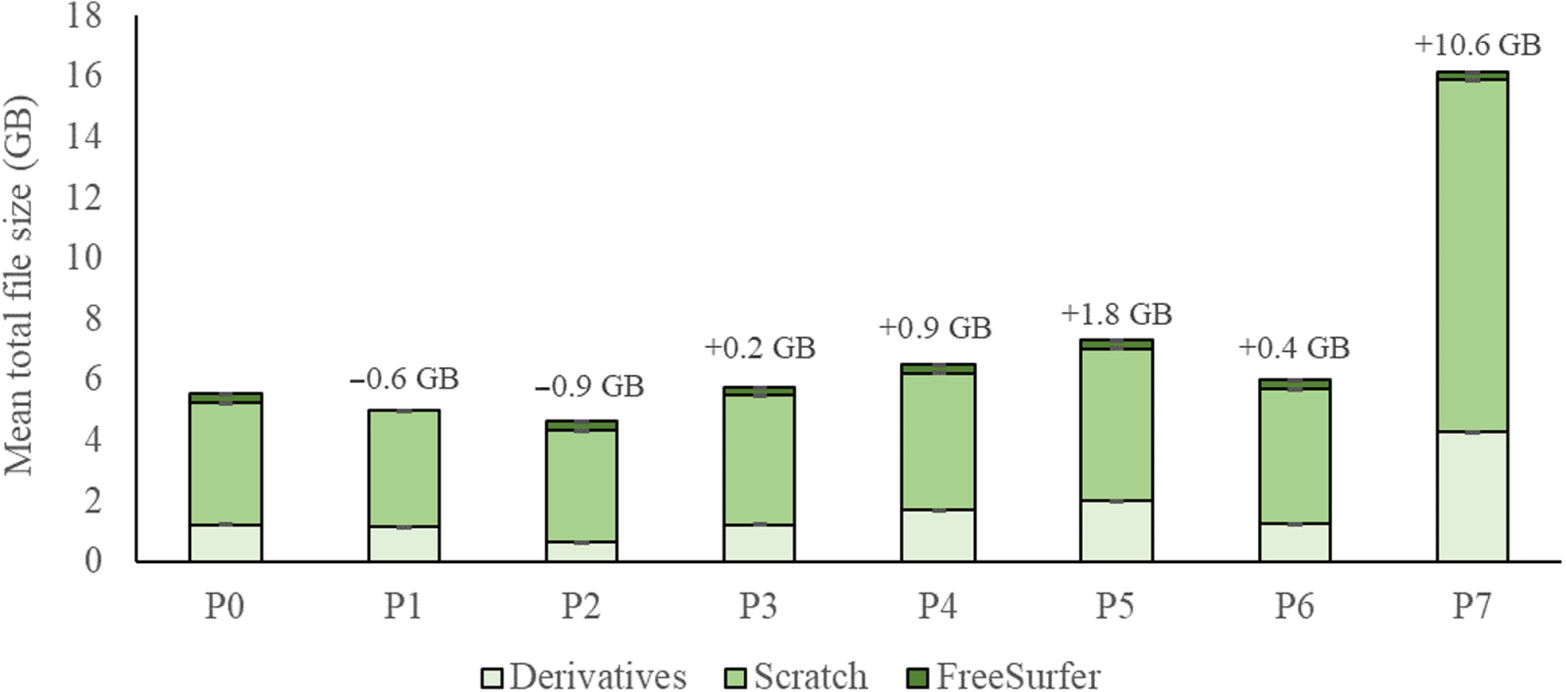
The mean total size (GB) of all files generated for a given subject, split by ‘derivatives’ (final output files), ‘scratch’ (working directory), and the subject‐specific ‘FreeSurfer’ directory, for each pipeline. Error bars are one standard error of the mean, these are too small to be visible. The mean change in size relative to the baseline pipeline (P0) is highlighted for each experimental pipeline. *N* = 257. GB, gigabytes.

The mean carbon emissions of each pipeline are plotted against preprocessing performance (task activation and smoothness), duration, and energy usage in Figure 3. The mean file size for each pipeline is in Figure 4. Table 3 provides results of repeated measures ANOVAs used to observe the effect of pipeline identity on each dependent variable.

**TABLE 3:** Main effects of pipeline for each dependent variable, for both frequentist and Bayesian ANOVAs.

Category	Dependent variable	Frequentist ANOVA	Bayesian ANOVA
Computing metrics	Carbon emissions	*F* (3.6, 857.4) = 4121.7, *p* < .001*, *η*_p_^2^ = .95	BF_10_ = ∞ Extreme evidence
	Duration	*F* (4.1, 989.3) = 2929.0, *p* < .001*, *η*_p_^2^ = .92	BF_10_ = ∞ Extreme evidence
	CPU energy	*F* (4.8, 1151.7) = 3222.8, *p* < .001*, *η*_p_^2^ = .93	BF_10_ = ∞ Extreme evidence
	RAM energy	*F* (2.5, 594.1) = 5628.1, *p* < .001*, *η*_p_^2^ = .96	BF_10_ = ∞ Extreme evidence
Smoothness	Pre‐smoothing	*F* (6, 1524) = 3885.9, *p* < .001*, *η*_p_^2^ = .94	BF_10_ = ∞ Extreme evidence
	Post‐smoothing	*F* (7, 1757) = 5081.5, *p* < .001*, *η*_p_^2^ = .95	BF10 = ∞ Extreme evidence
Task activation	Left primary motor cortex	*F* (7, 1778) = 19.2, *p* < .001*, *η*_p_^2^ = .07	BF_10_ = 3.235 × 10^21^ Extreme evidence
	Pre‐SMA	*F* (7, 1764) = 15.7, *p* < .001*, *η*_p_^2^ = .06	BF_10_ = 5.848 × 10^16^ Extreme evidence
Total file size		*F* (1.1, 270.2) = 124,366.2, *p* < .001*, *η*_p_^2^ = .998	BF_10_ = ∞ Extreme evidence

*Note*: * reflects significant results at *p* < .05. Due to violation of the assumption of sphericity, Greenhouse–Geisser correction applied to ANOVAs for carbon emissions, duration, CPU energy, and RAM energy, and total file size. It was not possible to perform sphericity tests for smoothness or task activation ANOVAs given singular SSP matrices (see Section 2.5). *N* = 257.

Abbreviations: CPU, central processing unit; pre‐SMA, pre‐supplementary motor area; RAM, random‐access memory; SSP, sum‐of‐squares‐and‐products.

For each pipeline, effects for each metric relative to baseline (determined by contrasts in Table S5) are parsed below. Given that carbon emissions are exactly proportional to overall energy usage,^4^ CPU and RAM energy usage are only discussed when their patterns of significance deviate from those of emissions. Here, reductions in energy usage are typically attributable to reduced compute time. As such, percent changes in emissions and duration are typically comparable.

#### 3.1.1 Pipeline 1—No FreeSurfer surface reconstruction

Disabling FreeSurfer surface reconstruction reduced emissions and duration by 48% and 49%, respectively. This had no significant effect on statistical sensitivity, and even yielded decreases of 2% and 0.4% in pre‐smoothed and post‐smoothed smoothness, respectively.^5^ File size decreased by 10%.

#### 3.1.2 Pipeline 2—‘Sloppy’ testing mode

Enabling ‘sloppy’ mode (simplified registration) reduced emissions and duration by 44% and 41%, respectively. Reported activation in the pre‐SMA decreased by 10%, and pre‐smoothed smoothness increased by 5%. These performance losses were anticipated, given this mode is intended only for testing. File size decreased by 17%.

#### 3.1.3 Pipeline 3—Low memory mode

Low memory mode, which reduces memory usage at the cost of increasing disk usage in the working directory, reduced both emissions and duration by 6%. This had no impact on preprocessing performance (preprocessed data was identical to baseline). File size increased by 4%. Note that this memory optimisation appears to occur only during the compression of BOLD data. As such, savings in compute are likely to depend on the specific data being used.

#### 3.1.4 Pipeline 4—Adding a surface output space

Surprisingly, adding a surface output space *reduced* emissions and duration, by 4% and 5%, respectively (driven by a 4% decrease in CPU energy, but no significant change in RAM energy). Inclusion of a surface space may reduce redundancies in such a way that increases efficiency. Given that analysis was not performed on the generated surface space, there were no changes in preprocessing performance. File size increased by 17%. While this setting did reduce emissions, we do not recommend it as a route to doing so, given that other settings reduce emissions to a greater degree, and the cause of these reductions is clearer.

#### 3.1.5 Pipeline 5—Implementing ICA‐AROMA

Adding ICA‐AROMA had no significant impact on duration, but increased emissions by 3%. Reported activation in the pre‐SMA increased by 6%, with no significant change in the primary motor cortex. The mechanisms by which ICA‐AROMA may improve performance is beyond the scope of the current analysis, but is discussed in prior papers (e.g., Pruim et al., 2015). Post‐smoothing smoothness increased by 13%—to be expected given that AROMA entails a 6 mm smoothing kernel, higher than the 5 mm kernel applied to other pipelines. File size increased by 32%. Note that as of version 23.1.0, ICA‐AROMA has been removed from the fMRIPrep workflow due to imminent compatibility issues (https://github.com/nipreps/fmriprep/issues/2936).

#### 3.1.6 Pipeline 6—Implementing fieldmap‐free distortion correction

Fieldmap‐free distortion correction, intended for use in the absence of fieldmaps, increased both emissions and duration by 22%. This coincided with *decreases* in reported activation: 28% for the primary motor cortex and 19% for the pre‐SMA. Despite pre‐smoothed smoothness decreasing by 4%, post‐smoothed smoothness increased by 2%. File size increased by 8%. At least in fMRIPrep version 22.1.1., this tool appears to yield losses in performance. At time of writing, efforts from fMRIPrep developers to diagnose and remedy increases in distortion resulting from distortion correction are ongoing.^6^

#### 3.1.7 Pipeline 7—Increasing output space resolution

Increasing volumetric output space resolution increased emissions and duration by 70% and 14%, respectively. This particularly large increase in emissions was driven by a 224% increase in RAM energy, consistent with a substantial increase in file size of 191%. This was the only pipeline for which RAM energy exceeded CPU energy (see Figure 3d). This manipulation had no significant effect on statistical sensitivity.^7^ However, this pipeline decreased both pre‐ and post‐smoothed smoothness, by 3% and 2%, respectively.

### 3.2 Visualising anatomical variability and specificity

As in Esteban et al. (2019), we visually represented the anatomical specificity and variability of each pipeline. Activation count maps are presented below, to provide an illustration of qualitative consistency across pipelines. For the sake of brevity, other figures referenced below are in Supplementary Section ‘Visualising variability and specificity’. All relevant maps are on Neurovault (https://neurovault.org/collections/JNAGJRUI). In each case, maps for P3 (low memory) and P4 (added surface output space) are identical to P0 (baseline), as these manipulations did not affect fMRIPrep output.

**FIGURE 5:**
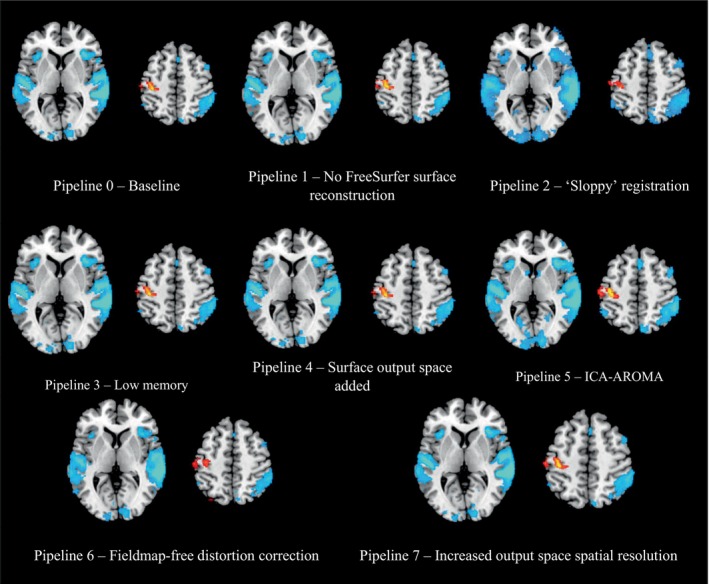
Activation count maps for each fMRIPrep pipeline. Values reflect the percentage of participants within the sample (*N* = 257) showing significant individual‐level activation in a given voxel for both the ‘go > successful stop’ (motor; hot colours) and ‘successful stop > go’ (response inhibition; cool colours) contrasts. Slices presented are at MNI coordinates *Z* = 4 and *Z* = 52. AROMA, automatic removal of motion artifacts; ICA, independent components analysis.

Activation count maps (Figure 5)^8^ reflect the percentage of subjects showing significant individual‐level activation in each voxel for both the ‘go > successful stop’ and ‘successful stop > go’ contrasts, thresholded at 5%. Similar qualitative patterns emerged across pipelines. Quantitatively (Table S6), P1 (no FreeSurfer surface reconstruction) and P6 (fieldmap‐free distortion correction) reduced the size of thresholded maps relative to P0. P5 (ICA‐AROMA) and P7 (increased output space resolution) led to reliable increases, while P2 (sloppy preprocessing) was inconsistent. Similar patterns were observed for thresholded group‐level activation maps (Figure S8).

Timeseries SD maps (Figure S9) reflect the sample's mean SD of preprocessed timeseries values for each voxel across all 184 fMRI volumes. Variation was consistently highest around the brain outline and in anterior temporal/orbitofrontal regions—areas susceptible to distortion (Olman et al., 2009; Stenger, 2006). Particularly high variation around the outline was observed for P2, likely due to poor registration, and for P6, likely due to increased distortion. P6 also had 3% lower average SD than P0 (Table S7), perhaps reflecting poor sensitivity to statistical effects.

Activation SD maps (Figure S10) reflect the sample's mean SD of z‐statistics in the unthresholded contrast of ‘go > successful stop’ for each voxel. Qualitatively, variation was consistently highest in areas implicated in the go or successful stop trials, including left primary motor cortex, bilateral auditory cortex, and lateral temporoparietal regions. P5 had 3% higher SD than P0, while P6 was 5% lower (Table S7), likely reflecting increased and decreased statistical sensitivity, respectively.

## 4 DISCUSSION

Neuroimaging research frequently relies on computationally expensive data preprocessing. Here, we investigated the carbon footprint of preprocessing pipeline fMRIPrep and demonstrated that decreases in emissions do not necessitate reductions in preprocessing performance, as reflected in sensitivity to task activation and data smoothness. Box 1 provides recommendations based on these findings.

BOX 1 **Summary of recommendations for reducing the carbon footprint of fMRIPrep**

**Table:** 

Disable FreeSurfer surface reconstruction (‐‐fs‐no‐reconall) if reconstruction files are not required. This can almost halve one's emissions with no trade‐offs in performance. Disabling FreeSurfer surface reconstruction by default would be an impactful change to the software. Only when testing the feasibility of a pipeline, use ‘sloppy’ registration (‐‐sloppy). This should not be used in actual preprocessing—it can almost halve one's emissions but yields performance losses. Implement low memory mode (‐‐low‐mem). This can modestly reduce emissions while not impacting performance. Given that optimisation occurs during the compression of BOLD data, specific savings will depend on the data being used. Implement ICA‐AROMA (‐‐use‐aroma) if needed. This modestly increases carbon emissions but can benefit sensitivity to statistical activation (Although note that as of version 23.1.0, ICA‐AROMA has been removed from the fMRIPrep workflow due to imminent compatibility issues [https://github.com/nipreps/fmriprep/issues/2936]). At fMRIPrep version 22.1.1, the fieldmap‐free distortion correction technique (‐‐use‐syn‐sdc) can both increase emissions and degrade data quality, based on the data preprocessed here. Though not tested here, the use of scan‐specific fieldmaps, when available, is likely to be preferable. Refrain from using high‐resolution volumetric output space templates (‐‐output‐spaces MNI152NLin6Asym:res‐ < number>) unless necessary. This drastically increases emissions and storage usage, while not benefitting task sensitivity and only somewhat benefitting smoothness.

Although these recommendations are particularly relevant to neuroimaging, this paper can serve as a model to identify evidenced‐based recommendations for mitigating the carbon footprint of digital tools. Systematically studying the effect of parameters on emissions allows identification of acceptable levels of trade‐offs in performance. This has been applied to deep learning, where reductions in dataset size and energy consumption can be made with minimal performance losses (e.g., Bakhtiarifard et al., 2024; Li & Chao, 2021). We encourage researchers both inside and outside neuroimaging to integrate carbon tracking functions (e.g., CodeCarbon [Goyal‐Kamal et al., 2021], Carbontracker [Anthony et al., 2020]) into software, or use online calculators such as Green Algorithms (Lannelongue et al., 2021) or server‐side tools such as GA4HPC (https://www.green-algorithms.org/GA4HPC). This will enable researchers to measure carbon emissions and identify routes to reducing them. Tool developers could help here by integrating embedded carbon trackers and turning them on by default – for instance, automatic application of CodeCarbon in fMRIPrep. However, as demonstrated in the Supplementary Section ‘Complications using CodeCarbon’, this tool encounters issues such as with hardware isolation in the context of HPC Testing embedded carbon trackers on a wider variety of hardware and settings will help validation, and end users will benefit from advice on their appropriate application.

There are factors besides data processing parameters that impact the carbon footprint of computing. This includes the time of day that analysis is run—carbon intensity of local energy sources is highest at times of peak usage, at the start and end of the working day (Souter et al., 2023). By scheduling jobs to run at periods of low intensity (see CATS; https://github.com/GreenScheduler/cats), one could use the same amount of energy while decreasing emissions. One can also consider data storage, which has a carbon footprint due to server overheads. Our baseline pipeline produced an average of 5.55 GB per subject. Several pipelines increased file size, almost threefold for P7. Researchers can reduce this by removing ‘junk’ files, which for fMRIPrep constitutes up to 96% of the total size (see fMRIPrepCleanup to automate this; https://github.com/NickESouter/fMRIPrepCleanup). fMRIPrep users can use this information in conjunction with the above recommendations to further reduce their footprint. At time of writing, fMRIPrep developers are working to implement changes in the tool that would facilitate reduced preprocessing runtime and subsequent file size (see https://hackmd.io/@effigies/HyZ1GPHfp).

Although the energy needed for research computing likely makes up a small fraction of global CO_2_ emissions, this is an aspect of their work of which many researchers will have maximal control and knowledge needed to reduce their individual footprint. As the energy required for increasingly complex computing may continue to grow in the coming decades, it is important for individual researchers and tool developers to practice being mindful of their use of computing resources. This effort will be aided by transparency in reporting, using ‘Environmental impact statements’ (Souter et al., 2023). For instance, across all subjects and pipelines, preprocessing for the current study required 23,757 h of compute time and emitted an estimated 72.9 kg of CO_2_, equivalent to driving 419 miles (674 km) in a passenger car.^9^ Computing occurred in the United Kindom, with carbon intensity of 193.38 gCO_2_/kWh. Resulting emissions would have been higher in more carbon intensive countries, some of which have carbon intensity up to 4.5× that of the United Kingdom, and lower in others (Country Specific Electricity Grid Greenhouse Gas Emission Factors, 2022). Furthermore, we processed data for one 6‐min fMRI task—experiments containing multiple runs would result in a larger footprint per subject.

A neuroimaging researcher's computing footprint may quickly add up over the course of their career. Speculatively, if running the baseline fMRIPrep pipeline employed here on the same dataset (257 subjects, 6 min of task data), a hypothetical researcher would emit ~7.5 kgCO_2_e. Commonly, more than one fMRI task or run may be taken per study, perhaps with a total duration of 30 minutes, increasing carbon emissions fivefold (37.5 kgCO_2_e). As revisions of analysis plans or mistakes in preprocessing arise, this researcher may need to run preprocessing three times over for a single dataset (112.5 kgCO_2_e). Assuming this researcher analysed a dataset of this size once every 5 years over a 30 year career, this would amount to 675 kgCO_2_e, equivalent to three return flights from London to Paris as estimated by the Travel Carbon Footprint Calculator (https://travel-footprint-calculator.irap.omp.eu/). If another hypothetical researcher was using the same 6‐min task protocol and preprocessing approach used here but for a large population‐level dataset, such as the UK Biobank which currently includes task fMRI data for ~60,000 participants (https://www.ukbiobank.ac.uk; Miller et al., 2016), preprocessing in fMRIPrep for this sample would amount to 1751 kgCO_2_e, equivalent to 92% of a return flight from London to New York City. In this case, disabling surface reconstruction (a reduction of 48%) would reduce one's footprint by 840 kgCO_2_e. These are only hypothetical speculations, but demonstrate how research computing may have real impact over the course of multiple studies or a single large project. A comprehensive analysis of the carbon footprint of practices such as data collection, data analysis, and conference travel would provide a valuable contribution in allowing researchers to more precisely understand the relative contributions of each (see Epp et al. (2023) for analysis of the footprint of conference travel).

Best practice for minimising the footprint of research computing may depend on the dataset in question. In cases where a given dataset will be analysed once and never shared or reused, reducing compute by disabling unnecessary steps (e.g., switching off FreeSurfer reconstruction when not required^10^) may be the best route. If shared and used by others, however, there may be environmental benefits of providing more derivatives rather than fewer. For example, if runtime was halved by removing FreeSurfer, and two secondary researchers then independently run FreeSurfer on this same dataset, there would be a likely net increase in energy consumption. The public sharing and re‐use of high‐quality preprocessed datasets with comprehensive derivatives (including surface reconstruction files, for example) could be beneficial in increasing the likelihood that individual researchers will not need to re‐preprocess data. However, any potential savings should be weighed against the carbon footprint of storing public data on cloud computing services (Souter et al., 2023). In this sense, sharing a relatively small number of high‐quality datasets is likely to be more sustainable than many labs sharing a large number of lower‐quality ones (e.g., smaller sample size). Striking this balance in the most effective way will require a continued research culture shift to sharing of data that is findable, accessible, interoperable, and reusable (Wilkinson et al., 2016). The carbon footprint of sharing and downloading different data types through cloud computing services is relevant here, and quantification of this footprint would a valuable contribution. However, this is currently challenging given there may not be full transparency from commercial cloud computing providers on carbon emissions arising from their services.

There are limitations to the current study. First, to quantify preprocessing performance, we used statistical task sensitivity and mean data smoothness, similar to metrics used by Esteban et al. (2019) when comparing fMRIPrep to FSL FEAT. Task activation is limited in so far as we cannot assume ‘ground truth’ activation against which to benchmark performance. We relied on the assumption that a sufficiently robust paradigm should yield predictable hotspots. Other methods for quantifying preprocessing performance exist, such as split‐half resampling (Churchill et al., 2015). Second, while carbon tracking tools have been validated as providing reasonable estimates (Jay et al., 2023), they are not perfect. Measures of energy usage rely on the proxy of TDP, which can underestimate usage (Lannelongue et al., 2023). Relatedly, our energy usage calculations do not account for idle power consumption of the HPC system that would be indirectly attributable to submission of our jobs. This decision was made in order to avoid emissions estimates being biased by the number of other jobs, including those from other users, running on the system. However, in real terms, this means the energy usage attributable to fMRIPrep is likely somewhat underestimated. Third, carbon emission values reported are specific to the hardware employed in our institutional HPC cluster. Certain effects may be more or less evident on different systems. Fourth, we did not use live carbon intensity data in estimating emissions (see https://carbonintensity.org.uk). Comparison of data across pipelines would otherwise have been confounded, given that carbon intensity varies systematically by time of day and counterbalancing job start time would have been impractical. Fifth, this study does not consider environmental effects beyond electricity, such as water use and hardware manufacturing. Sixth, the analysis conducted here is specific to the output generated by a single version of fMRIPrep (22.1.1.). Future changes to the software (including those which may be specifically aimed at increasing environmental sustainability, such as efficiency of surface reconstruction) may affect both the quality of the output and the required computational resources. The current results should be interpreted within this context. Finally, this study focused on individual efforts to make research computing more environmentally sustainable. While these efforts are vital, institutional system‐level changes in policy and hardware efficiency will also be needed to facilitate change.

In conclusion, we have provided evidence‐based recommendations for how neuroimaging researchers can reduce the carbon footprint of fMRIPrep, without sacrificing data quality. We encourage similar investigations of other digital data processing and analysis tools.

## 5 ENVIRONMENTAL IMPACT STATEMENT

Across all subjects and pipelines, preprocessing for the current experiment in fMRIPrep produced an estimated 72.9 kg of carbon dioxide equivalent emissions (kgCO_2_eq), as determined using an in‐house server‐side carbon tracker. Computing was conducted in the southeast of England, with estimated annual average carbon intensity of 193.38 g of CO_2_ per kilowatt hour (http://www.carbonfootprint.com).

## AUTHOR CONTRIBUTIONS

**Nicholas E. Souter:** Conceptualisation; data curation; formal analysis; investigation; methodology; project administration; software; visualisation; writing—original draft. **Nikhil Bhagwat:** Software; writing—review and editing. **Chris Racey:** Methodology; writing—review and editing. **Reese Wilkinson:** Methodology; software; validation; writing—review and editing. **Niall W. Duncan:** Writing—review and editing. **Gabrielle Samuel:** Funding acquisition; writing—review and editing. **Loïc Lannelongue:** Methodology; validation; writing—review and editing. **Raghavendra Selvan:** Conceptualisation; funding acquisition; writing—review and editing. **Charlotte L. Rae:** Conceptualisation; funding acquisition; methodology; writing—review and editing.

## CONFLICT OF INTEREST STATEMENT

The authors have no competing interests or conflicts of interest to disclose.

## Supporting information


**Data S1:** Supporting information.

## Data Availability

Outcome data for each pipeline (performance and carbon tracking metrics) are available on the Open Science Framework (OSF; https://osf.io/4e26k). Group‐level fMRI maps are on Neurovault (https://neurovault.org/collections/JNAGJRUI). All code used to run fMRIPrep, perform carbon tracking, and process the resulting data is available and documented on GitHub (https://github.com/NickESouter/fMRIPrep-Carbon-Footprint).

## Footnotes

^1^
The only exception being for the supplementary P8 in which parallelisation was increased. Jobs for the pipeline were necessarily run on nodes using more efficient processors, creating a hardware confound for this pipeline. See supplementary section ‘Parallelisation analysis’ for more detail.

^2^
PUE will vary based on the time of year at which it is measured. Estimated emissions for the computing conducted here may be higher at other institutions, given that 1.28 is considerably lower than the PUE industry standard of 1.55, as reported for 2022 (Davis et al., 2022).

^3^
It is worth noting that the actual EVs provided in the public dataset linked to Gorgolewski et al. (2017) do not conform to the design described in the paper. Each fixed duration is 1 second from the onset, accounting for the fixation cross and only half (500 ms) of the decision period. We have opted not to replicate this approach because (a) it would reduce statistical power by removing part of the trial from the model and (b) changing to the intended duration of 1.5 seconds should serve only to change the magnitude of the effect relative to the original dataset, rather than its qualitative nature (i.e., location of peak activation).

^4^
Emissions were estimated by multiplying energy usage by average carbon intensity for the UK and power usage effectiveness of the University of Sussex HPC architecture.

^5^
While disabling surface reconstruction does not itself impact the outcome of volumetric preprocessing, doing so in fMRIPrep changes the use of registration tool from bbregister (FreeSurfer) to FLIRT (FSL), leading to changes in the output file.

^6^

https://github.com/nipreps/fmriprep/issues/3013; https://github.com/nipreps/sdcflows/pull/391.

^7^
Conflicting results were found for supplementary ROIs: −6% in auditory cortex and + 8% in insula.

^8^
Activation count maps for supplementary pipelines P8 and P9 are in Supplementary Figure 7.

^9^
Assuming 108.1 gCO_2_/km, as taken from the 2022 average estimate of new passenger cars in Europe: https://www.eea.europa.eu/en/analysis/indicators/co2-performance-of-new-passenger.

^10^
Use of analysis involving surface reconstruction files could theoretically increase statistical power, facilitating a smaller sample size. It is currently unclear how the extent of reduction in sample size (and therefore computing demand) would balance against savings from not running surface reconstruction.
