# Supplementary material for: Measuring and reducing the carbon footprint of fMRI preprocessing in fMRIPrep
Source: Hum Brain Mapp. 2024 Aug 26;45(12):e70003. doi: 10.1002/hbm.70003 (PMC11345634; doi:10.1002/hbm.70003)
Supplement: Supplementary file 1 — Data S1: Supporting information. [file HBM-45-e70003-s001.docx]

Measuring and reducing the carbon footprint of fMRI preprocessing in fMRIPrep

Supplementary Materials

Nicholas E. Souter ^a^, Nikhil Bhagwat ^b^, Chris Racey ^a, c^, Reese Wilkinson ^d^, Niall W. Duncan ^e^, Gabrielle Samuel ^f^, Loïc Lannelongue ^g, h, i, j^, Raghavendra Selvan ^k, l^, Charlotte L. Rae ^a^

^a^ School of Psychology, University of Sussex, Brighton, United Kingdom

^b^ McConnell Brain Imaging Centre, The Neuro (Montreal Neurological Institute - Hospital), McGill University; Montreal, Quebec, Canada

^c^ Sussex Neuroscience, University of Sussex, Brighton, United Kingdom

^d^ Department of Physics and Astronomy, University of Sussex, Brighton BN1 9QH, UK

^e^ Graduate Institute of Mind, Brain and Consciousness, Taipei Medical University, Taipei, Taiwan

^f^ Department of Global Health and Social Medicine, King’s College London, London, United Kingdom

^g^ Cambridge Baker Systems Genomics Initiative, Department of Public Health and Primary Care, University of Cambridge, Cambridge, United Kingdom

^h^ British Heart Foundation Cardiovascular Epidemiology Unit, Department of Public Health and Primary Care, University of Cambridge, Cambridge, United Kingdom

^i^ Victor Phillip Dahdaleh Heart and Lung Research Institute, University of Cambridge, Cambridge, United Kingdom

^j^ Health Data Research UK Cambridge, Wellcome Genome Campus and University of Cambridge, Cambridge, United Kingdom

^k^ Department of Computer Science, University of Copenhagen, 2100, Copenhagen, Denmark

^l^ Department of Neuroscience, University of Copenhagen, 2200, Copenhagen, Denmark

# Deviations from preregistration

This project was preregistered on the OSF (<https://osf.io/839pa>) on March 31^st^, 2023. This preregistration was created using the Psychological Research Preregistration-Quantitative (PRP-QUANT) Template, version 2 (available at [https://www.psycharchives.org](https://www.psycharchives.org/)).

Deviations were made from this preregistration. (1) We devised 9 pipelines that would be included in analysis, including the 7 described in this paper, as well as two manipulating the parallelisation of preprocessing jobs through manipulation of the ‘nthreads’ fMRIPrep flag. Ultimately, we were not able to confidently make conclusions regarding parallelisation due to the presence of complicating factors. Description and analysis of these two pipelines have therefore been moved to the supplementary ‘*Parallelisation Analysis*’ section, including discussion of the relevant complicating factors. (2) We planned to only extract one contrast for the stop signal task in FSL FEAT; go > successful stop. The reverse of this contrast would be extracted merely by multiplying z-values by -1. In fact, it was necessary to also extract the contrast of successful stop > go in FEAT, in order to generate and analyse relevant thresholded individual-level activation maps for standard deviation maps. (3) Estimates of carbon emissions, duration of preprocessing, and measures of energy usage were intended to be extracted from CodeCarbon, which is integrated with the fMRIPrep command line. Due to complications with our server architecture (detailed in the methods section and supplementary materials), this was not feasible, and a server-side tool (analogous to GA4HPC; <https://www.green-algorithms.org/GA4HPC>) was used instead. (4) To ease interpretation, duration of preprocessing for each pipeline is reported in hours, rather than in seconds as originally registered. This did not affect statistical analysis of this variable. (5) When conducting post-hoc contrasts we planned to compare each pipeline in a stepwise manner within each dependent variable (e.g., comparing the least carbon intensive to the second least, which is in turn compared to the third least, etc.). To ease interpretation of results, we have compared each experimental pipeline to the baseline (Pipeline 0), with false discovery rate correction applied. (6) The numeric ID given to each pipeline has been updated from the pre-registration, such that pipelines are ordered from lowest to highest mean carbon emissions. This was done to ease interpretation of data in Figure 3. (7) We had originally stated that disabling FreeSurfer surface reconstruction was “*Predicted to decrease both carbon emissions and preprocessing performance, possibly to unacceptable levels*.” We have since appreciated that removal of surface reconstruction alone should have no impact on volumetric preprocessing, and the stated prediction for this pipeline has therefore been updated accordingly. (8) Finally, although not discussed in the preregistration, mean total file size has been added as a dependent variable for each pipeline, given the relevance of this metric to the carbon footprint of computing.

# Parallelisation Analysis

## Background

In our [preregistration](https://osf.io/839pa), we had planned to manipulate the fMRIPrep flag ‘nthreads’. According to the [fMRIPrep usage page](https://fmriprep.org/en/stable/usage.html) this flag manipulates the “*Maximum number of threads across all processes*”. Changing this flag was expected to affect the computational resources provided to each processing job, which was in turn predicted to impact the amount of compute used for preprocessing. The baseline pipeline (P0) and all main paper experimental pipelines used an nthreads value of 5. As shown in Supplementary Table 1, we planned a variant (P8) in which parallelisation was increased to 16 threads, and another (P9) in which parallelisation was removed, down to 1 thread. We present comparisons of these pipelines to P0 below. As covered in the following ‘*Discussion*’ section, we opted to make these analyses supplementary due to complications in the interpretation of results.

### Supplementary Table 1. Description of supplementary parallelisation fMRIPrep pipeline variants

| ID | Label | Flag addition | Description & predicted impact |
| --- | --- | --- | --- |
| 8 | Increase parallelisation | --nthreads **16** | - Increases the number of threads used across all processes, up from a default of 5. Predicted to increase carbon emissions while reducing runtime, and having no impact on preprocessing performance. |
| 9 | Remove parallelisation | --nthreads **1** | - Decreases the number of threads used across all processes, down from a default of 5. Predicted to decrease carbon emissions while increasing runtime, and having no effect on preprocessing performance. |

Note: ‘flag addition’ refers to the respective arguments passed to the fMRIPrep command line (<https://fmriprep.org/en/stable/usage.html>). Values or strings given to arguments are in bold.

## Results

Below, for P0, P8, and P9, we present mean values for preprocessing performance metrics and energy usage/duration against emissions (Supplementary Figure 1) and the average size of files generated for a given subject (Supplementary Figure 2). Supplementary Table 2 provides frequentist and Bayesian repeated measures ANOVAs for each dependent variable across these three pipelines, using the same structure as for analysis in the main paper. Significant main effects are parsed by separately comparing P8 and P9 to P0, in Supplementary Table 2. Interpretation of both pipelines follows.

| 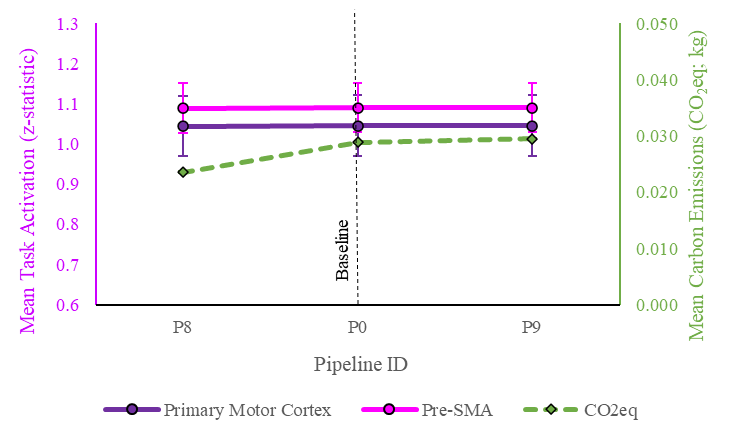 | 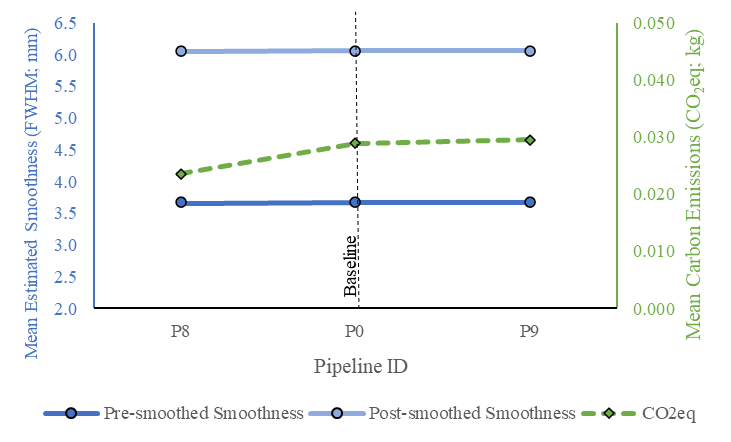 | P0 – Baseline  P8 – Increased parallelisation  P9 – Removed parallelisation |
| --- | --- | --- |
| 1. Mean statistical task activation (z-statistic) | 1. Mean estimated smoothness (mm) |  |
| 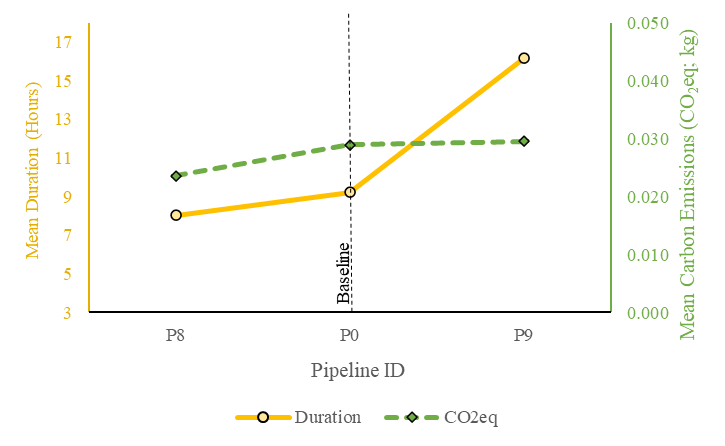 | 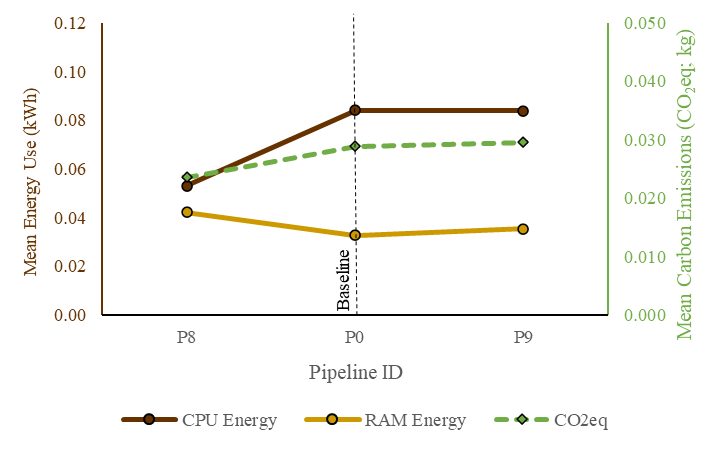 |  |
| 1. Mean pipeline duration (hours) | 1. Mean energy use for CPU and RAM (kWh) |  |

### *Supplementary Figure 1. Estimated carbon emissions (dotted green line) plotted against (a) statistical task activation in regions of interest, (b) estimated data smoothness, (c) duration of preprocessing, and (d) CPU and RAM energy usage, for the baseline pipeline and both supplementary parallelisation pipelines. Error bars reflect one standard error of the mean. These are frequently too small to be visible. Note that the scale used varies between variables, see text below for percent changes. N = 257. Pre-SMA = pre-supplementary motor area, CPU = central processing unit, RAM = random-access memory, CO2eq = carbon dioxide equivalent, mm = millimetres, kWh = kilowatt hours, kg = kilograms*

| 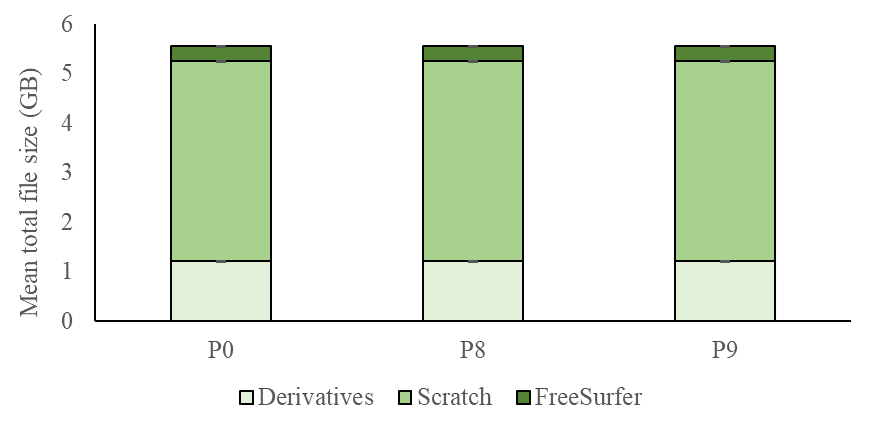 |
| --- |

### *Supplementary Figure 2. The mean total size (GB) of all files generated for a given subject, split by ‘derivatives’ (final output files), ‘scratch’ (working directory), and the subject-specific ‘FreeSurfer’ directory, for the baseline pipeline and both supplementary parallelisation pipelines. Error bars are one standard error of the mean, these are too small to be visible. N = 257*

### Supplementary Table 2. Main effects of pipeline for each dependent variable, for both frequentist and Bayesian ANOVAs, considering the baseline pipeline and both supplementary parallelisation pipelines

| Category | Dependent variable | Frequentist ANOVA | Bayesian ANOVA |
| --- | --- | --- | --- |
| Computing metrics | Carbon emissions | F(2, 506) = 421.4, *p* < .001*, η_p_^2^ = .63 | BF_10_ = 4.986×10^116^ Extreme evidence |
|  | Duration | F(1.5, 387.0) = 3978.6, *p* < .001*, η_p_^2^ = .94 | BF_10_ = ∞  Extreme evidence |
|  | CPU energy | F(1.9, 487.7) = 1586.0, *p* < .001*, η_p_^2^ = .86 | BF_10_ = 3.748×10^264^ Extreme evidence |
|  | RAM energy | F(2.0, 499.4) = 461.0, *p* < .001*, η_p_^2^ = .65 | BF_10_ = 1.257x10^124^ Extreme evidence |
| Smoothness | Pre-smoothing | F(2, 512) = 1.7, *p* = .191, η_p_^2^ = .01 | N/A |
|  | Post-smoothing | F(2, 510) = 0.1, *p* = .938, η_p_^2^ < .01 | N/A |
| Task activation | Left primary motor cortex | F(2, 512) < 0.1, *p* = .982, η_p_^2^ < .01 | N/A |
|  | Pre-SMA | F(2, 510) = 0.7, *p* = .495, η_p_^2^ < .01 | N/A |
| Total file size | | F(1.0, 255.0) = 1.2, *p* = .277, η_p_^2^ = .01 | N/A |

Note: * reflects significant results at *p* < .05. Due to violation of the assumption of sphericity, Greenhouse-Geisser correction applied to ANOVAs for carbon emissions, duration, CPU energy, and RAM energy, and total file size. It was not possible to perform sphericity tests for smoothness or task activation ANOVAs given singular SSP matrices – see *Online methods* section 4.5 for more detail. It was not possible to calculate Bayes factors for smoothness, task activation, or file size in JASP, possibly due to identical values across pipelines. N = 257. CPU = central processing unit, RAM = random-access memory, pre-SMA = pre-supplementary motor area.

### Supplementary Table 3. Planned contrasts for all significant supplementary parallelisation frequentist ANOVAs, with each pipeline compared to P0 (baseline)

| Pipeline | Mean percent difference from P0 | Result | Pipeline | Mean percent difference from P0 | Result |
| --- | --- | --- | --- | --- | --- |
| *Carbon emissions* | | | *Duration* | | |
| **P8** | **-18.3%** | **t(506) = 23.5, *p* < .001*** | **P8** | **-12.9%** | **t(500) = 12.1, *p* < .001*** |
| **P9** | **+2.3%** | **t(506) = -3.0, *p* = .003*** | **P9** | **+75.0%** | **t(500) = -70.5, *p* < .001*** |
| *CPU energy use* | | | *RAM energy use* | | |
| **P8** | **-36.7%** | **t(506) = 48.9, *p* < .001*** | **P8** | **+28.8%** | **t(502) = -29.4, *p* < .001*** |
| P9 | -0.1% | t(506) = 0.2, *p* = .812 | **P9** | **+8.0%** | **t(502) = -8.3, *p* < .001*** |

Note: * reflects significant results at p < .05. Significant results are also in bold. N = 257.

### *Pipeline 8 – Increasing parallelisation*

Increasing parallelisation from 5 threads up to 16 reduced emissions and duration by 18% and 13%, respectively. This change in emissions was driven by a decrease in CPU energy of 37%, but an *increase* in RAM energy of 29%. Increasing parallelisation fractionally impacted the reproducibility of preprocessing but did not significantly alter any performance metric from baseline (no change of more than 0.05%). The activation count map, timeseries standard deviation (SD) map, and activation SD map for P8 (see supplementary section *‘Visualising variability and specificity*’) deviated slightly from P0, but these changes were negligible.

### *Pipeline 9 – Removing parallelisation*

Removing parallelisation, down from 5 threads to 1 thread, increased emissions by only 2%, while increasing duration by 75%. While there was no significant change in CPU energy usage, RAM energy use increased by 8%. This pipeline had no impact on task activation or smoothness, producing identical output to baseline. The activation count map, timeseries standard deviation (SD) map, and activation SD map for P9 (see supplementary section *‘Visualising variability and specificity*’) were identical to P0.

## Discussion

Following analysis of P8 and P9, it became clear that certain methodological issues and remaining questions complicated their comparison to P0.

First, it is unclear why duration of preprocessing when removing parallelisation (P9) increased drastically despite modest changes in energy usage and therefore estimated carbon emissions. As for all other pipelines, one would expect these variables to increase or decrease proportionally. This may be in part attributable to a misunderstanding of how computational resources are being used in this case. In accordance with [documentation for the University of Sussex high-performance computing cluster](https://docs.hpc.sussex.ac.uk/apollo2/resources.html#id1) (using a Sun Grid Engine), in order to facilitate the use of multiple threads/slots within a programme such as fMRIPrep, it was necessary to manipulate the ‘openmp’ flag upon job submission to facilitate parallel environments. As such, we set openmp to be equal to the value used for nthreads (1, 5, or 16) for each pipeline. To interrogate the unexpected dissociation in emissions and duration, ten subjects were re-run through fMRIPrep, this time retaining an openmp value of 5 but decreasing nthreads to 1, as in P9. As seen in Supplementary Figure 3a, doing so reduced emissions relative to P0 by 11% and increased duration by 53% (rather than increasing emissions by 2% and increasing duration by 75%, as was found for P9). This alludes to a pattern whereby removing parallelisation may use less energy at the cost of slowing preprocessing. This comparison demonstrates that effects of varying openmp and nthreads dissociate in important ways that we do not completely understand. All main paper pipelines kept both values consistently at 5, but manipulations of both variables in P8 and P9 may produce unfair comparisons.

Second, unforeseen computational restrictions on our high-performance computing architecture produced an important hardware confound when comparing increased parallelisation (P8) to baseline (P0). As noted in the main paper, all main paper pipelines were run on nodes with an Intel® Xeon® Processor E5-2640 v3. It was not possible to use these nodes for P8, given that they were not able to support jobs utilising 16 threads. As such, jobs for this pipeline were automatically spread over other Intel® Xeon® CPU processors including Gold 6240R (N = 49), Gold 5115 (N = 139), Gold 6226R (N = 20), and Gold 5215 (N = 49). The nodes used for P8 were therefore inherently more efficient than those used for all other pipelines. To attempt to quantify the extent of this confound, ten subjects were re-run on Gold 5115 processors, but keeping nthreads and openmp at 5 rather than increasing to 16. These data were compared to all data from P0 (N = 257) on E5-2640 processors and all data from P8 derived from Gold 5115 processors (N = 139). As seen in Supplementary Figure 3b, relative to P0, emissions decreased by 26% and duration decreased by 9%, merely by switching to a more efficient processor. These savings are therefore in fact greater than when using the same processors with nthreads and openmp of 16 (12% decrease for emissions, 8% decrease for duration). This paints a picture whereby increased parallelisation increases emissions, rather than decreasing them as suggested by P8. Evidently, the extent of this hardware confound complicates any direct comparison between P0 and P8. Given that Sussex HPC users are unable to request 16 slots on E5-2640 processors due to memory constraints, it was not feasible to re-run P8 jobs on our default processors and remove this confound. While all other pipelines could theoretically be rerun on more efficient nodes using Gold 5115 processors, for instance, we did not judge the extra compute and runtime required to be worth this adjustment.

Given this range of complications, confounds, and uncertainties, we judged that it would not be wise to make concrete recommendations relating to the use of nthreads in fMRIPrep. Beyond the factors discussed here, any specific effects of manipulating parallelisation are likely to be largely dependent on a user’s available hardware and HPC architecture. Neuroimagers wishing to use HPC resources as efficiently as possible should consult technicians and/or IT personnel familiar with their own institutional architecture.

| 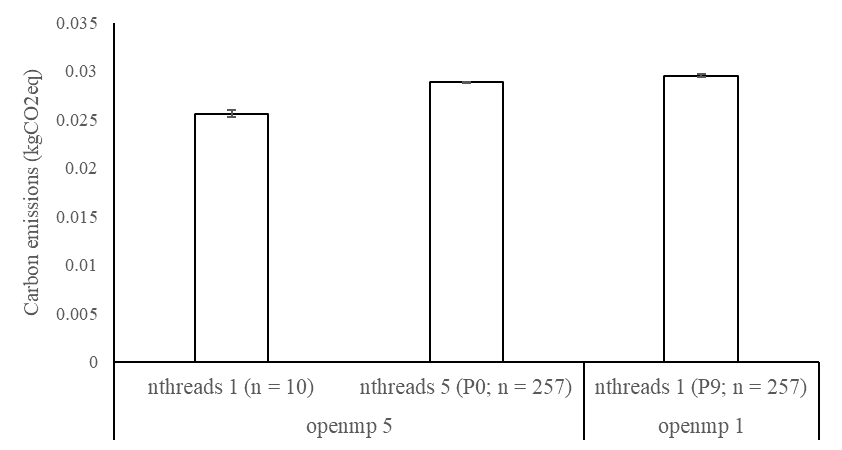 | 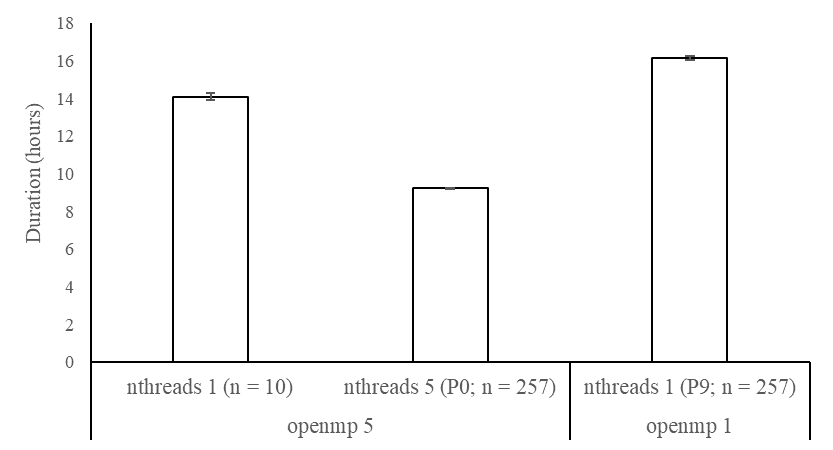 |
| --- | --- |
| (a) Dissociable influences of nthreads and openmp | |
| 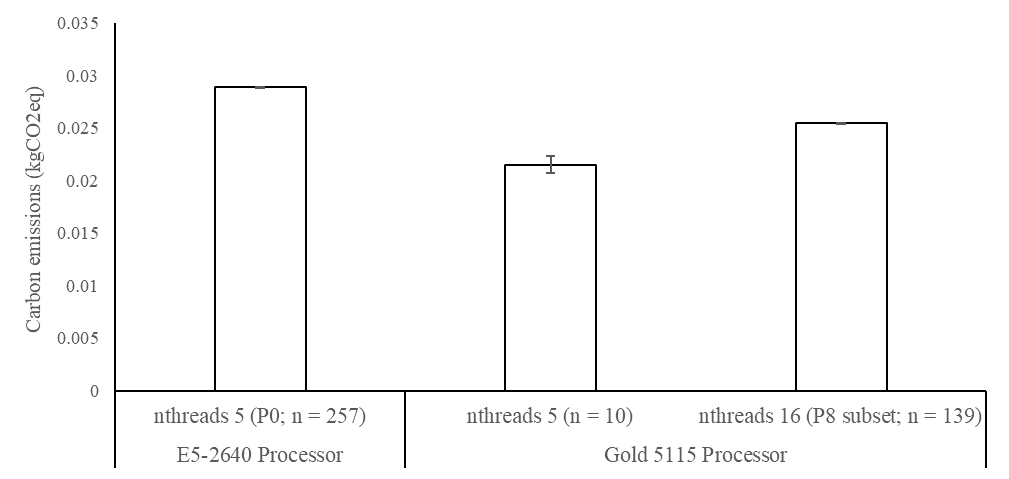 | 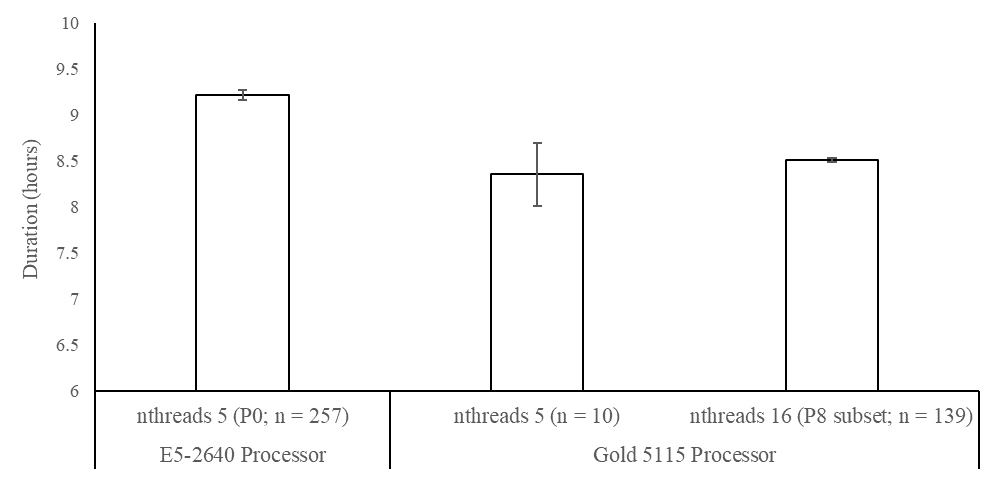 |
| (b) Accounting for hardware confounds in pipeline 8 | |

### *Supplementary Figure 3. Follow-up investigations of complications with supplementary parallelisation analysis. This includes (a) evidence of dissociable influences of the nthreads and openmp flag, originally assumed to be equivalent, for both carbon emissions and duration, and (b) adjustment for the processor hardware confound present for pipeline 8, for both carbon emissions and duration*

# fMRIPrep Citation Boilerplates

Results included in this manuscript come from preprocessing performed using fMRIPrep 22.1.1 (Esteban et al. (2018; 2019); RRID:SCR_016216), which is based on Nipype 1.8.5 (K. Gorgolewski et al. (2011); K. J. Gorgolewski et al. (2018); RRID:SCR_002502).

Many internal operations of *fMRIPrep* use *Nilearn* 0.9.1 (Abraham et al. 2014, RRID:SCR_001362), mostly within the functional processing workflow. For more details of the pipeline, see [the section corresponding to workflows in *fMRIPrep*’s documentation](https://fmriprep.readthedocs.io/en/latest/workflows.html).

The [below] boilerplate text was automatically generated by fMRIPrep with the express intention that users should copy and paste this text into their manuscripts *unchanged*. It is released under the [CC0](https://creativecommons.org/publicdomain/zero/1.0/) license.

## Pipeline 0 – Baseline

**Anatomical data preprocessing**

A total of 1 T1-weighted (T1w) images were found within the input BIDS dataset. The T1-weighted (T1w) image was corrected for intensity non-uniformity (INU) with N4BiasFieldCorrection (Tustison et al. 2010), distributed with ANTs 2.3.3 (Avants et al. 2008, RRID:SCR_004757), and used as T1w-reference throughout the workflow. The T1w-reference was then skull-stripped with a Nipype implementation of the antsBrainExtraction.sh workflow (from ANTs), using OASIS30ANTs as target template. Brain tissue segmentation of cerebrospinal fluid (CSF), white-matter (WM) and gray-matter (GM) was performed on the brain-extracted T1w using fast (FSL 6.0.5.1:57b01774, RRID:SCR_002823, Zhang, Brady, and Smith 2001). Brain surfaces were reconstructed using recon-all (FreeSurfer 7.2.0, RRID:SCR_001847, Dale, Fischl, and Sereno 1999), and the brain mask estimated previously was refined with a custom variation of the method to reconcile ANTs-derived and FreeSurfer-derived segmentations of the cortical gray-matter of Mindboggle (RRID:SCR_002438, Klein et al. 2017). Volume-based spatial normalization to two standard spaces (MNI152NLin6Asym, MNI152NLin2009cAsym) was performed through nonlinear registration with antsRegistration (ANTs 2.3.3), using brain-extracted versions of both T1w reference and the T1w template. The following templates were selected for spatial normalization: FSL’s MNI ICBM 152 non-linear 6th Generation Asymmetric Average Brain Stereotaxic Registration Model [Evans et al. (2012), RRID:SCR_002823; TemplateFlow ID: MNI152NLin6Asym], ICBM 152 Nonlinear Asymmetrical template version 2009c [Fonov et al. (2009), RRID:SCR_008796; TemplateFlow ID: MNI152NLin2009cAsym].

**Functional data preprocessing**

For each of the 1 BOLD run found per subject (across all tasks and sessions), the following preprocessing was performed. First, a reference volume and its skull-stripped version were generated using a custom methodology of fMRIPrep. Head-motion parameters with respect to the BOLD reference (transformation matrices, and six corresponding rotation and translation parameters) are estimated before any spatiotemporal filtering using mcflirt (FSL 6.0.5.1:57b01774, Jenkinson et al. 2002). The BOLD time-series (including slice-timing correction when applied) were resampled onto their original, native space by applying the transforms to correct for head-motion. These resampled BOLD time-series will be referred to as preprocessed BOLD in original space, or just preprocessed BOLD. The BOLD reference was then co-registered to the T1w reference using bbregister (FreeSurfer) which implements boundary-based registration (Greve and Fischl 2009). Co-registration was configured with six degrees of freedom. Several confounding time-series were calculated based on the preprocessed BOLD: framewise displacement (FD), DVARS and three region-wise global signals. FD was computed using two formulations following Power (absolute sum of relative motions, Power et al. (2014)) and Jenkinson (relative root mean square displacement between affines, Jenkinson et al. (2002)). FD and DVARS are calculated for each functional run, both using their implementations in Nipype (following the definitions by Power et al. 2014). The three global signals are extracted within the CSF, the WM, and the whole-brain masks. Additionally, a set of physiological regressors were extracted to allow for component-based noise correction (CompCor, Behzadi et al. 2007). Principal components are estimated after high-pass filtering the preprocessed BOLD time-series (using a discrete cosine filter with 128s cut-off) for the two CompCor variants: temporal (tCompCor) and anatomical (aCompCor). tCompCor components are then calculated from the top 2% variable voxels within the brain mask. For aCompCor, three probabilistic masks (CSF, WM and combined CSF+WM) are generated in anatomical space. The implementation differs from that of Behzadi et al. in that instead of eroding the masks by 2 pixels on BOLD space, a mask of pixels that likely contain a volume fraction of GM is subtracted from the aCompCor masks. This mask is obtained by dilating a GM mask extracted from the FreeSurfer’s aseg segmentation, and it ensures components are not extracted from voxels containing a minimal fraction of GM. Finally, these masks are resampled into BOLD space and binarized by thresholding at 0.99 (as in the original implementation). Components are also calculated separately within the WM and CSF masks. For each CompCor decomposition, the k components with the largest singular values are retained, such that the retained components’ time series are sufficient to explain 50 percent of variance across the nuisance mask (CSF, WM, combined, or temporal). The remaining components are dropped from consideration. The head-motion estimates calculated in the correction step were also placed within the corresponding confounds file. The confound time series derived from head motion estimates and global signals were expanded with the inclusion of temporal derivatives and quadratic terms for each (Satterthwaite et al. 2013). Frames that exceeded a threshold of 0.5 mm FD or 1.5 standardized DVARS were annotated as motion outliers. Additional nuisance timeseries are calculated by means of principal components analysis of the signal found within a thin band (crown) of voxels around the edge of the brain, as proposed by (Patriat, Reynolds, and Birn 2017). The BOLD time-series were resampled into standard space, generating a preprocessed BOLD run in MNI152NLin6Asym space. First, a reference volume and its skull-stripped version were generated using a custom methodology of fMRIPrep. All resamplings can be performed with a single interpolation step by composing all the pertinent transformations (i.e. head-motion transform matrices, susceptibility distortion correction when available, and co-registrations to anatomical and output spaces). Gridded (volumetric) resamplings were performed using antsApplyTransforms (ANTs), configured with Lanczos interpolation to minimize the smoothing effects of other kernels (Lanczos 1964). Non-gridded (surface) resamplings were performed using mri_vol2surf (FreeSurfer).

## Pipeline 1 – No FreeSurfer surface reconstruction

**Anatomical data preprocessing**

A total of 1 T1-weighted (T1w) images were found within the input BIDS dataset. The T1-weighted (T1w) image was corrected for intensity non-uniformity (INU) with N4BiasFieldCorrection (Tustison et al. 2010), distributed with ANTs 2.3.3 (Avants et al. 2008, RRID:SCR_004757), and used as T1w-reference throughout the workflow. The T1w-reference was then skull-stripped with a Nipype implementation of the antsBrainExtraction.sh workflow (from ANTs), using OASIS30ANTs as target template. Brain tissue segmentation of cerebrospinal fluid (CSF), white-matter (WM) and gray-matter (GM) was performed on the brain-extracted T1w using fast (FSL 6.0.5.1:57b01774, RRID:SCR_002823, Zhang, Brady, and Smith 2001). Volume-based spatial normalization to two standard spaces (MNI152NLin6Asym, MNI152NLin2009cAsym) was performed through nonlinear registration with antsRegistration (ANTs 2.3.3), using brain-extracted versions of both T1w reference and the T1w template. The following templates were selected for spatial normalization: FSL’s MNI ICBM 152 non-linear 6th Generation Asymmetric Average Brain Stereotaxic Registration Model [Evans et al. (2012), RRID:SCR_002823; TemplateFlow ID: MNI152NLin6Asym], ICBM 152 Nonlinear Asymmetrical template version 2009c [Fonov et al. (2009), RRID:SCR_008796; TemplateFlow ID: MNI152NLin2009cAsym].

**Functional data preprocessing**

*As in Pipeline 0*

## Pipeline 2 – ‘Sloppy’ testing mode

**Anatomical data preprocessing**

*As in Pipeline 0*

**Functional data preprocessing**

*As in Pipeline 0*

## Pipeline 3 – Low memory mode

**Anatomical data preprocessing**

*As in Pipeline 0*

**Functional data preprocessing**

*As in Pipeline 0*

## Pipeline 4 – Adding a surface output space

**Anatomical data preprocessing**

*As in Pipeline 0*

**Functional data preprocessing**

For each of the 1 BOLD run found per subject (across all tasks and sessions), the following preprocessing was performed. First, a reference volume and its skull-stripped version were generated using a custom methodology of *fMRIPrep*. Head-motion parameters with respect to the BOLD reference (transformation matrices, and six corresponding rotation and translation parameters) are estimated before any spatiotemporal filtering using mcflirt (FSL 6.0.5.1:57b01774, Jenkinson et al. 2002). The BOLD time-series (including slice-timing correction when applied) were resampled onto their original, native space by applying the transforms to correct for head-motion. These resampled BOLD time-series will be referred to as *preprocessed BOLD in original space*, or just *preprocessed BOLD*. The BOLD reference was then co-registered to the T1w reference using bbregister (FreeSurfer) which implements boundary-based registration (Greve and Fischl 2009). Co-registration was configured with six degrees of freedom. Several confounding time-series were calculated based on the *preprocessed BOLD*: framewise displacement (FD), DVARS and three region-wise global signals. FD was computed using two formulations following Power (absolute sum of relative motions, Power et al. (2014)) and Jenkinson (relative root mean square displacement between affines, Jenkinson et al. (2002)). FD and DVARS are calculated for each functional run, both using their implementations in *Nipype* (following the definitions by Power et al. 2014). The three global signals are extracted within the CSF, the WM, and the whole-brain masks. Additionally, a set of physiological regressors were extracted to allow for component-based noise correction (*CompCor*, Behzadi et al. 2007). Principal components are estimated after high-pass filtering the *preprocessed BOLD* time-series (using a discrete cosine filter with 128s cut-off) for the two *CompCor* variants: temporal (tCompCor) and anatomical (aCompCor). tCompCor components are then calculated from the top 2% variable voxels within the brain mask. For aCompCor, three probabilistic masks (CSF, WM and combined CSF+WM) are generated in anatomical space. The implementation differs from that of Behzadi et al. in that instead of eroding the masks by 2 pixels on BOLD space, a mask of pixels that likely contain a volume fraction of GM is subtracted from the aCompCor masks. This mask is obtained by dilating a GM mask extracted from the FreeSurfer’s *aseg* segmentation, and it ensures components are not extracted from voxels containing a minimal fraction of GM. Finally, these masks are resampled into BOLD space and binarized by thresholding at 0.99 (as in the original implementation). Components are also calculated separately within the WM and CSF masks. For each CompCor decomposition, the *k* components with the largest singular values are retained, such that the retained components’ time series are sufficient to explain 50 percent of variance across the nuisance mask (CSF, WM, combined, or temporal). The remaining components are dropped from consideration. The head-motion estimates calculated in the correction step were also placed within the corresponding confounds file. The confound time series derived from head motion estimates and global signals were expanded with the inclusion of temporal derivatives and quadratic terms for each (Satterthwaite et al. 2013). Frames that exceeded a threshold of 0.5 mm FD or 1.5 standardized DVARS were annotated as motion outliers. Additional nuisance timeseries are calculated by means of principal components analysis of the signal found within a thin band (*crown*) of voxels around the edge of the brain, as proposed by (Patriat, Reynolds, and Birn 2017). The BOLD time-series were resampled into standard space, generating a *preprocessed BOLD run in MNI152NLin6Asym space*. First, a reference volume and its skull-stripped version were generated using a custom methodology of *fMRIPrep*. The BOLD time-series were resampled onto the following surfaces (FreeSurfer reconstruction nomenclature): *fsaverage*. All resamplings can be performed with *a single interpolation step* by composing all the pertinent transformations (i.e. head-motion transform matrices, susceptibility distortion correction when available, and co-registrations to anatomical and output spaces). Gridded (volumetric) resamplings were performed using antsApplyTransforms (ANTs), configured with Lanczos interpolation to minimize the smoothing effects of other kernels (Lanczos 1964). Non-gridded (surface) resamplings were performed using mri_vol2surf (FreeSurfer).

## Pipeline 5 – Implementing ICA-AROMA

**Anatomical data preprocessing**

*As in Pipeline 0*

**Functional data preprocessing**

For each of the 1 BOLD run found per subject (across all tasks and sessions), the following preprocessing was performed. First, a reference volume and its skull-stripped version were generated using a custom methodology of *fMRIPrep*. Head-motion parameters with respect to the BOLD reference (transformation matrices, and six corresponding rotation and translation parameters) are estimated before any spatiotemporal filtering using mcflirt (FSL 6.0.5.1:57b01774, Jenkinson et al. 2002). The BOLD time-series (including slice-timing correction when applied) were resampled onto their original, native space by applying the transforms to correct for head-motion. These resampled BOLD time-series will be referred to as *preprocessed BOLD in original space*, or just *preprocessed BOLD*. The BOLD reference was then co-registered to the T1w reference using bbregister (FreeSurfer) which implements boundary-based registration (Greve and Fischl 2009). Co-registration was configured with six degrees of freedom. Several confounding time-series were calculated based on the *preprocessed BOLD*: framewise displacement (FD), DVARS and three region-wise global signals. FD was computed using two formulations following Power (absolute sum of relative motions, Power et al. (2014)) and Jenkinson (relative root mean square displacement between affines, Jenkinson et al. (2002)). FD and DVARS are calculated for each functional run, both using their implementations in *Nipype* (following the definitions by Power et al. 2014). The three global signals are extracted within the CSF, the WM, and the whole-brain masks. Additionally, a set of physiological regressors were extracted to allow for component-based noise correction (*CompCor*, Behzadi et al. 2007). Principal components are estimated after high-pass filtering the *preprocessed BOLD* time-series (using a discrete cosine filter with 128s cut-off) for the two *CompCor* variants: temporal (tCompCor) and anatomical (aCompCor). tCompCor components are then calculated from the top 2% variable voxels within the brain mask. For aCompCor, three probabilistic masks (CSF, WM and combined CSF+WM) are generated in anatomical space. The implementation differs from that of Behzadi et al. in that instead of eroding the masks by 2 pixels on BOLD space, a mask of pixels that likely contain a volume fraction of GM is subtracted from the aCompCor masks. This mask is obtained by dilating a GM mask extracted from the FreeSurfer’s *aseg* segmentation, and it ensures components are not extracted from voxels containing a minimal fraction of GM. Finally, these masks are resampled into BOLD space and binarized by thresholding at 0.99 (as in the original implementation). Components are also calculated separately within the WM and CSF masks. For each CompCor decomposition, the *k* components with the largest singular values are retained, such that the retained components’ time series are sufficient to explain 50 percent of variance across the nuisance mask (CSF, WM, combined, or temporal). The remaining components are dropped from consideration. The head-motion estimates calculated in the correction step were also placed within the corresponding confounds file. The confound time series derived from head motion estimates and global signals were expanded with the inclusion of temporal derivatives and quadratic terms for each (Satterthwaite et al. 2013). Frames that exceeded a threshold of 0.5 mm FD or 1.5 standardized DVARS were annotated as motion outliers. Additional nuisance timeseries are calculated by means of principal components analysis of the signal found within a thin band (*crown*) of voxels around the edge of the brain, as proposed by (Patriat, Reynolds, and Birn 2017). The BOLD time-series were resampled into standard space, generating a *preprocessed BOLD run in MNI152NLin6Asym space*. First, a reference volume and its skull-stripped version were generated using a custom methodology of *fMRIPrep*. Automatic removal of motion artifacts using independent component analysis (ICA-AROMA, Pruim et al. 2015) was performed on the *preprocessed BOLD on MNI space* time-series after removal of non-steady state volumes and spatial smoothing with an isotropic, Gaussian kernel of 6mm FWHM (full-width half-maximum). Corresponding “non-aggresively” denoised runs were produced after such smoothing. Additionally, the “aggressive” noise-regressors were collected and placed in the corresponding confounds file. All resamplings can be performed with *a single interpolation step* by composing all the pertinent transformations (i.e. head-motion transform matrices, susceptibility distortion correction when available, and co-registrations to anatomical and output spaces). Gridded (volumetric) resamplings were performed using antsApplyTransforms (ANTs), configured with Lanczos interpolation to minimize the smoothing effects of other kernels (Lanczos 1964). Non-gridded (surface) resamplings were performed using mri_vol2surf (FreeSurfer).

## Pipeline 6 – Implementing fieldmap-free distortion correction

**Anatomical data preprocessing**

*As in Pipeline 0*

**Preprocessing of B_0_ inhomogeneity mappings**

A total of 1 fieldmaps were found available within the input BIDS structure for this particular subject. A deformation field to correct for susceptibility distortions was estimated based on *fMRIPrep*’s *fieldmap-less* approach. The deformation field is that resulting from co-registering the EPI reference to the same-subject T1w-reference with its intensity inverted (Wang et al. 2017; Huntenburg 2014). Registration is performed with antsRegistration (ANTs 2.3.3), and the process regularized by constraining deformation to be nonzero only along the phase-encoding direction, and modulated with an average fieldmap template (Treiber et al. 2016).

**Functional data preprocessing**

For each of the 1 BOLD run found per subject (across all tasks and sessions), the following preprocessing was performed. First, a reference volume and its skull-stripped version were generated using a custom methodology of *fMRIPrep*. Head-motion parameters with respect to the BOLD reference (transformation matrices, and six corresponding rotation and translation parameters) are estimated before any spatiotemporal filtering using mcflirt (FSL 6.0.5.1:57b01774, Jenkinson et al. 2002). The estimated *fieldmap* was then aligned with rigid-registration to the target EPI (echo-planar imaging) reference run. The field coefficients were mapped on to the reference EPI using the transform. The BOLD reference was then co-registered to the T1w reference using bbregister (FreeSurfer) which implements boundary-based registration (Greve and Fischl 2009). Co-registration was configured with six degrees of freedom. Several confounding time-series were calculated based on the *preprocessed BOLD*: framewise displacement (FD), DVARS and three region-wise global signals. FD was computed using two formulations following Power (absolute sum of relative motions, Power et al. (2014)) and Jenkinson (relative root mean square displacement between affines, Jenkinson et al. (2002)). FD and DVARS are calculated for each functional run, both using their implementations in *Nipype* (following the definitions by Power et al. 2014). The three global signals are extracted within the CSF, the WM, and the whole-brain masks. Additionally, a set of physiological regressors were extracted to allow for component-based noise correction (*CompCor*, Behzadi et al. 2007). Principal components are estimated after high-pass filtering the *preprocessed BOLD* time-series (using a discrete cosine filter with 128s cut-off) for the two *CompCor* variants: temporal (tCompCor) and anatomical (aCompCor). tCompCor components are then calculated from the top 2% variable voxels within the brain mask. For aCompCor, three probabilistic masks (CSF, WM and combined CSF+WM) are generated in anatomical space. The implementation differs from that of Behzadi et al. in that instead of eroding the masks by 2 pixels on BOLD space, a mask of pixels that likely contain a volume fraction of GM is subtracted from the aCompCor masks. This mask is obtained by dilating a GM mask extracted from the FreeSurfer’s *aseg* segmentation, and it ensures components are not extracted from voxels containing a minimal fraction of GM. Finally, these masks are resampled into BOLD space and binarized by thresholding at 0.99 (as in the original implementation). Components are also calculated separately within the WM and CSF masks. For each CompCor decomposition, the *k* components with the largest singular values are retained, such that the retained components’ time series are sufficient to explain 50 percent of variance across the nuisance mask (CSF, WM, combined, or temporal). The remaining components are dropped from consideration. The head-motion estimates calculated in the correction step were also placed within the corresponding confounds file. The confound time series derived from head motion estimates and global signals were expanded with the inclusion of temporal derivatives and quadratic terms for each (Satterthwaite et al. 2013). Frames that exceeded a threshold of 0.5 mm FD or 1.5 standardized DVARS were annotated as motion outliers. Additional nuisance timeseries are calculated by means of principal components analysis of the signal found within a thin band (*crown*) of voxels around the edge of the brain, as proposed by (Patriat, Reynolds, and Birn 2017). The BOLD time-series were resampled into standard space, generating a *preprocessed BOLD run in MNI152NLin6Asym space*. First, a reference volume and its skull-stripped version were generated using a custom methodology of *fMRIPrep*. All resamplings can be performed with *a single interpolation step* by composing all the pertinent transformations (i.e. head-motion transform matrices, susceptibility distortion correction when available, and co-registrations to anatomical and output spaces). Gridded (volumetric) resamplings were performed using antsApplyTransforms (ANTs), configured with Lanczos interpolation to minimize the smoothing effects of other kernels (Lanczos 1964). Non-gridded (surface) resamplings were performed using mri_vol2surf (FreeSurfer).

## Pipeline 7 – Increasing output space resolution

**Anatomical data preprocessing**

*As in Pipeline 0*

**Functional data preprocessing**

*As in Pipeline 0*

## Pipeline 8 – Increasing parallelisation

**Anatomical data preprocessing**

*As in Pipeline 0*

**Functional data preprocessing**

*As in Pipeline 0*

## Pipeline 9 – Removing parallelisation

**Anatomical data preprocessing**

*As in Pipeline 0*

**Functional data preprocessing**

*As in Pipeline 0*

# Complications using CodeCarbon

As of version 22.1.0 of fMRIPrep (December 12th, 2022), the package CodeCarbon (Goyal-Kamal et al., 2021) has been integrated into the fMRIPrep command line, by author NB. This allows users to extract estimates of carbon emissions for an individual subject’s pipeline. Embedding carbon tracking packages into software is an important first step in understanding and reducing the carbon footprint of one’s computing. It should be noted, however, that we encountered complications when using CodeCarbon at the level of the University of Sussex high-performance computing (HPC) cluster. Namely, we observed systematic increases and decreases in reported energy consumption (and therefore emissions) according to the time at which a given fMRIPrep task (specific to a subject) was submitted. Supplementary Figure 4a provides a visualisation of this issue for one of our pipelines (Pipeline 2).

This was unlikely to be an issue with fMRIPrep itself. The issue persisted when running one individual subject ahead of all others within a pipeline, removing the possibility that this initial surge was the product of difficulty overwriting shared files within a given output directory. It also persisted when each subject was allocated an entirely separate output and working directory. Similarly, this was unlikely to be an issue with the ways in which tasks were being submitted to the cluster. Our default approach was to submit all 257 participants as individual tasks within a single array. Given user-specific restrictions on use, 30 subjects were able to simultaneously run at any one time. However, this issue also persisted when subjects were instead initiated as a series of independent jobs, rather than many tasks within a single job.

Further inspection of the code revealed that CodeCarbon appears to capture all CPU energy used by a given node that a task is using, including baseload. This is likely to become a problem when a task is sharing a node with multiple other tasks, some of which may be particularly resource intensive. For our purposes, taking exclusive use of a sufficient number of nodes would not have been feasible within our time frame (computing across all pipelines would have taken over two years to complete).

To further diagnose this issue, author RW developed an in-house tracking system that relies on retroactive use of HPC logs. Energy usage (kWh) for CPU was estimated by multiplying total CPU time by the power usage of a CPU core. The power usage of a CPU core was obtained by dividing the thermal design power (TDP; 90W) of the node in use by the total number of available CPU cores (16). Total CPU time was obtained from the task logs and represents the sum of the active time of each CPU core used. Energy usage for RAM (kWh) was estimated by multiplying maximum memory used (rounded up to the nearest GB) by volatile memory consumption (0.3725) and runtime. Estimated emissions (gCO_2_/kWh) were calculated by multiplying the estimated total energy usage (CPU + RAM) by 193.38, the average carbon intensity value for the UK provided in the 2022 v1.0 release of Country Specific Electricity Factors (2022) from [www.carbonfootprint.com](http://www.carbonfootprint.com), and by 1.28, the estimated power usage effectiveness (PUE) of the Sussex HPC architecture as determined by data centre baseline power readings measure on October 19th 2023^^[[1]](#footnote-1)^^. This approach is analogous to that used by the Green Algorithms server-side tool GA4HPC (<https://www.green-algorithms.org/GA4HPC>). Given that this code points to the task itself, rather than the node it is being executed on, this analysis is not sensitive to other activity on the cluster. Indeed, as seen in Supplementary Figure 4b, this code produces a plot of emissions that does not experience systematic increases or decreases over time.

Use of CodeCarbon is likely to be beneficial when users are submitting individual subjects for preprocessing, provided this job is not sharing a node with other jobs. In large scale preprocessing as employed here, it may be necessary to instead make use of HPC logs.

| 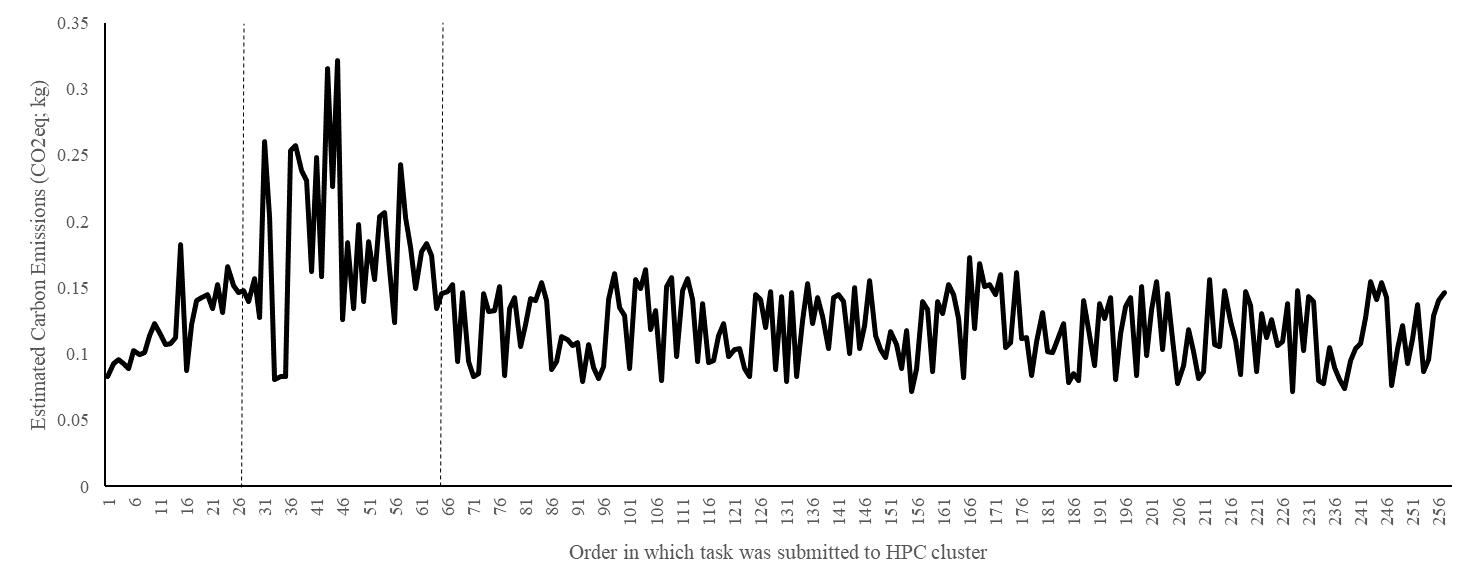 |
| --- |
| 1. – CodeCarbon |
| 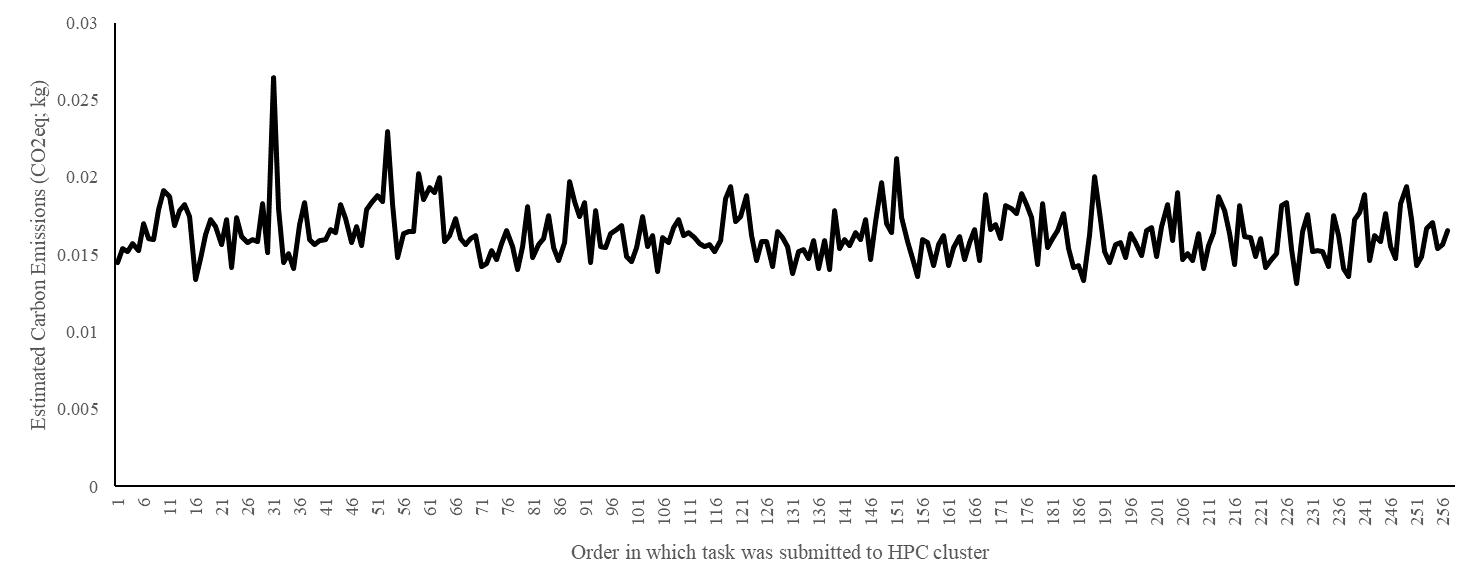 |
| 1. – HPC logs |

## *Supplementary Figure 4. The relationship between the order in which fMRIPrep tasks (subjects) for a given pipeline were submitted to the HPC cluster, and estimated carbon emissions as derived from (a) CodeCarbon as it built into fMRIPrep, and (b) measured retrospectively using HPC logs with original code. Estimates derived for the same jobs (fMRIPrep for Pipeline 2). Despite all tasks being allocated equivalent computational resources, this demonstrates systematic increases and decreases in estimated emissions when using CodeCarbon, due to baseload and presence of other users’ tasks. This is not observed when using HPC logs. HPC = high performance computing, CO2eq = carbon dioxide equivalent, kg = kilograms*

# Supplementary Task Activation

| 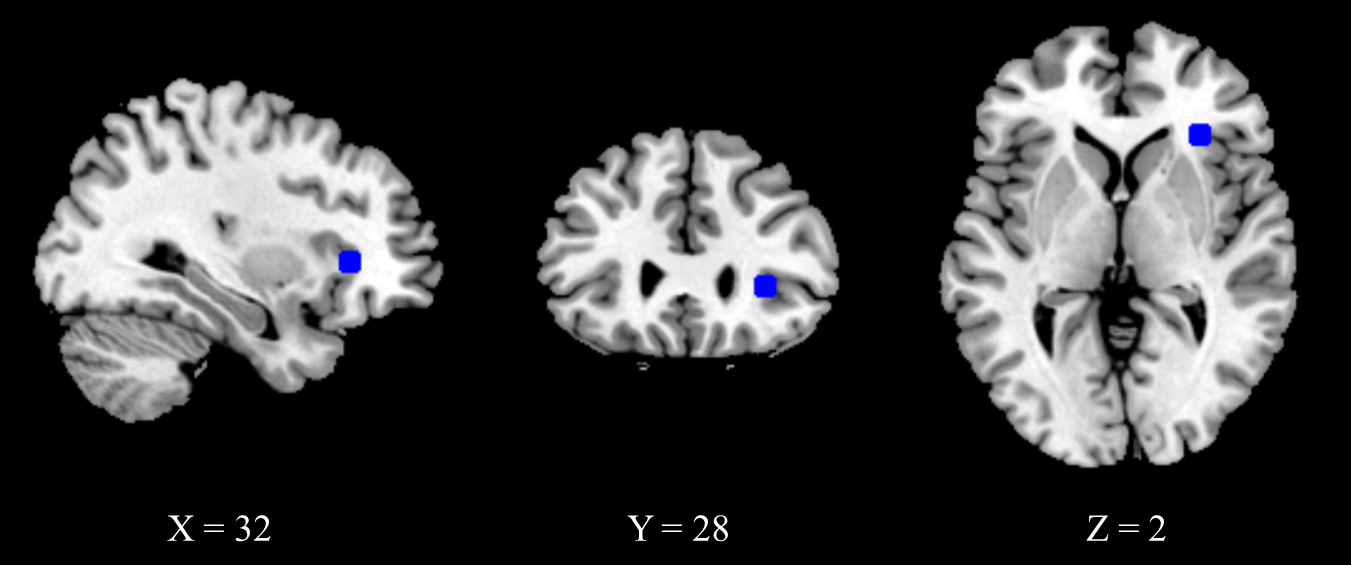 |
| --- |
| 1. – Response inhibition ROI (right insula) |
| 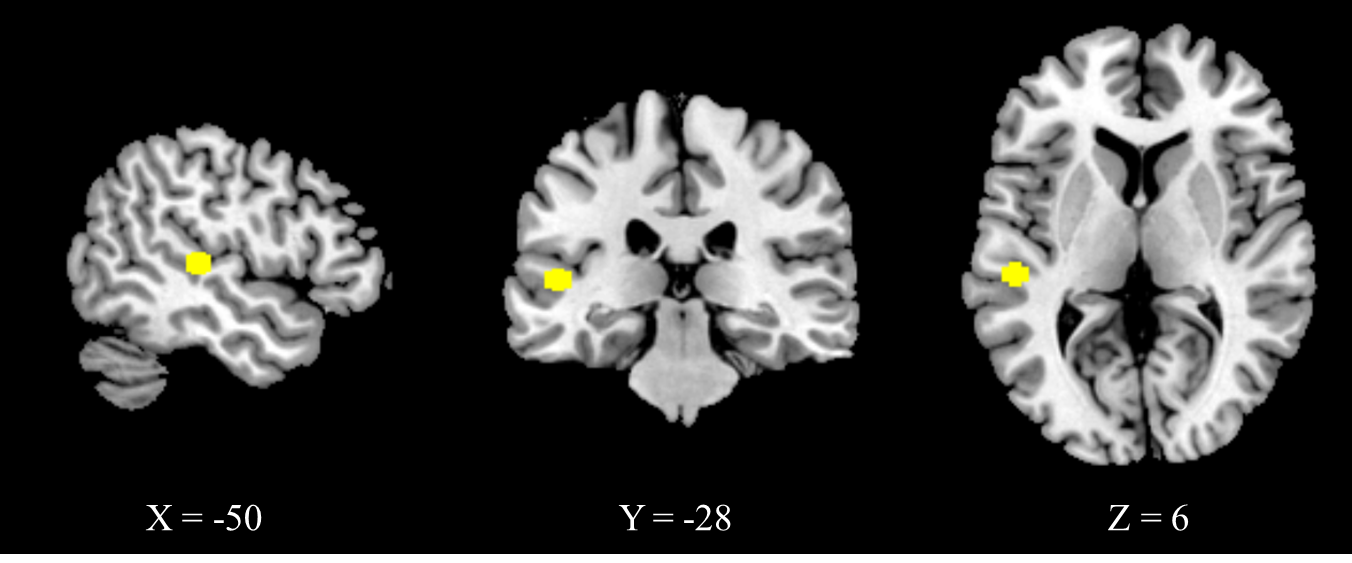 |
| 1. – Response inhibition ROI (left auditory cortex) |

## *Supplementary Figure 5. Supplementary ROIs for use in Featquery, including the right insula and left auditory cortex, for successful response inhibition. ROI = region of interest. Visualised using MRICron*

| 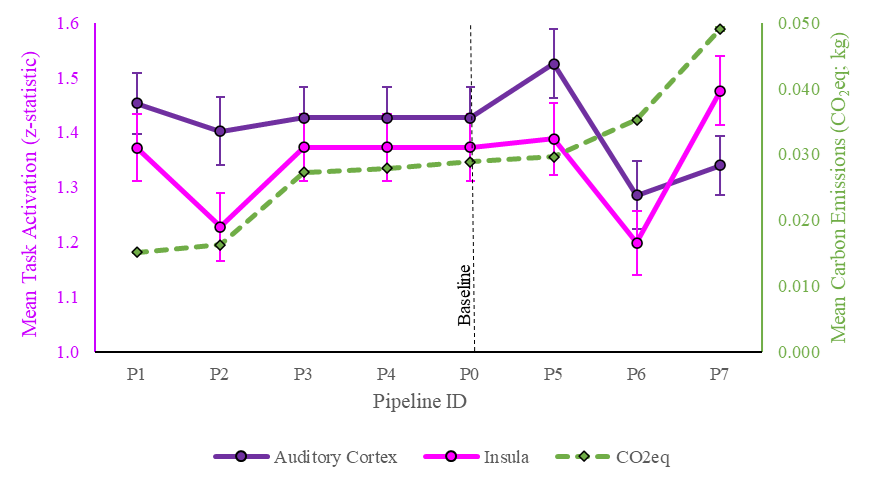 | **P0** – Baseline  **P1** – No FreeSurfer surface reconstruction  **P2** – ‘Sloppy’ registration  **P3** – Low memory  **P4** – Surface output space added  **P5** – ICA-AROMA  **P6** – Fieldmap-free distortion correction  **P7** – Increased output space spatial resolution |
| --- | --- |

## *Supplementary Figure 6. Estimated carbon emissions plotted against mean statistical task activation in supplementary regions of interest. Error bars reflect one standard error of the mean. For estimated emissions, these are too small to be visible. N = 257*

## Supplementary Table 4. Main effects of pipeline on supplementary task activation, for both frequentist and Bayesian ANOVAs

| Region | Frequentist ANOVA | Bayesian ANOVA |
| --- | --- | --- |
| **Right insula** | **F(7, 1785) = 18.4, *p* < .001*, η_p_^2^= .07** | BF_10_ = 2.558×10^20^, Extreme evidence |
| **Left auditory cortex** | **F(7, 1764) = 10.8, *p* < .001*, η_p_^2^= .04** | BF_10_ = 1.039×10^10^, Extreme evidence |

Note: * reflects significant results at *p* < .05. It was not possible to perform sphericity tests given singular SSP matrices – see *Online methods* section 4.5 for more detail. N = 257.

Contrasts for the frequentist ANOVAs are reported below in Supplementary Table 5 and are summarised here.

**Pipeline 1 – No FreeSurfer surface reconstruction**: Disabling surface reconstruction had no significant impact on activation in either region.

**Pipeline 2 – ‘Sloppy’ testing mode**: Implementing ‘sloppy’ mode significantly reduced activation in the insula by 10% but had no significant impact on the auditory cortex.

**Pipeline 3 – Low memory**: Low memory mode produced identical output to the baseline pipeline and as such had no significant impact on activation in either region.

**Pipeline 4** – **Adding a surface output space:** Analysis was performed on data in the volumetric space and as such this pipeline had no significant impact on activation in either region relative to baseline.

**Pipeline 5 – Implementing ICA-AROMA**: AROMA significantly increased activation in the auditory cortex by 7% but had no significant effect on activation in the insula.

**Pipeline 6 – Implementing fieldmap-free distortion correction**: This step significantly reduced activation in both the insula and auditory cortex, by 13% and 10%, respectively.

**Pipeline 7** – **Increasing output space resolution:** Increasing the volumetric output space from 2mm to 1mm resolution produced mixed results, significantly decreasing activation by 6% in the auditory cortex, and significantly increasing activation by 8% in the insula.

# Frequentist ANOVA Contrasts

Below are contrasts for each significant main effect of pipeline observed across all dependent variables for the main paper analysis. Each experimental pipeline (P1-P7) was compared to the baseline pipeline (P0). This results in seven t-tests per significant main effect, with false discovery rate (FDR) correction using the Benjamini-Hochberg method applied within each dependent variable. The mean percent difference from baseline is presented for each experimental pipeline/dependent variable.

## Supplementary Table 5. Planned contrasts for all frequentist ANOVAs, with each pipeline compared to P0 (baseline)

| Pipeline | Mean percent difference from P0 | Result | Pipeline | Mean percent difference from P0 | Result |
| --- | --- | --- | --- | --- | --- |
| *Carbon emissions* | | | *Duration* | | |
| **P1** | **-47.6%** | **t(1687) = 58.6, *p* < .001*** | **P1** | **-49.5%** | **t(1694) = 76.2, *p* < .001*** |
| **P2** | **-43.6%** | **t(1687) = 53.9, *p* < .001*** | **P2** | **-40.6%** | **t(1694) = 62.7, *p* < .001*** |
| **P3** | **-5.6%** | **t(1687) = 7.1, *p* < .001*** | **P3** | **-5.9%** | **t(1694) = 9.1, *p* < .001*** |
| **P4** | **-3.5%** | **t(1687) = 4.6, *p* < .001*** | **P4** | **-4.6%** | **t(1694) = 7.4, *p* < .001*** |
| **P5** | **+2.7%** | **t(1687) = -3.3, *p* = .001*** | P5 | +1.1% | t(1694) = -1.7, *p* = .091 |
| **P6** | **+22.2%** | **t(1687) = -27.4, *p* < .001*** | **P6** | **+22.2%** | **t(1694) = -34.3, *p* < .001*** |
| **P7** | **+69.9%** | **t(1687) = -85.2, *p* < .001*** | **P7** | **+13.6%** | **t(1694) = -20.5, *p* < .001*** |
| *CPU energy usage* | | | *RAM energy usage* | | |
| **P1** | **-45.9%** | **t(1694) = 79.1, *p* < .001*** | **P1** | **-52.2%** | **t(1659) = 32.1, *p* < .001*** |
| **P2** | **-45.5%** | **t(1694) = 78.7, *p* < .001*** | **P2** | **-39.1%** | **t(1659) = 24.1, *p* < .001*** |
| **P3** | **-5.1%** | **t(1694) = 8.6, *p* < .001*** | **P3** | **-7.4%** | **t(1659) = 4.7, *p* < .001*** |
| **P4** | **-3.9%** | **t(1694) = 7.1, *p* < .001*** | P4 | -2.8% | t(**1659**) = 1.8, *p* = .067 |
| **P5** | **+1.9%** | **t(1694) = -3.1, *p* = .002*** | **P5** | **+4.8%** | **t(1659) = -3.0, *p* = .002*** |
| **P6** | **+13.8%** | **t(1694) = -23.8, *p* < .001*** | **P6** | **+43.5%** | **t(1659) = -26.8, *p* < .001*** |
| **P7** | **+9.4%** | **t(1694) = -16.2, *p* < .001*** | **P7** | **+224.3%** | **t(1659) = -136.7, *p* < .001*** |
| *Pre-smoothed smoothness* | | | *Post-smoothed smoothness* | | |
| **P1** | **-1.9%** | **t(1524) = 29.7, *p* < .001*** | **P1** | **-0.4%** | **t(1757) = 4.2, *p* < .001*** |
| **P2** | **+4.9%** | **t(1524) = -75.7, *p* < .001*** | P2 | +0.1% | t(1757) = -0.6, *p* = .759 |
| P3 | 0% | t(1524) < 0.1, *p* = 1 | P3 | 0% | t(1757) < 0.1, *p* = 1 |
| P4 | 0% | t(1524) < 0.1, *p* = 1 | P4 | 0% | t(1757) < 0.1, *p* = 1 |
| P5 | N/A | N/A | **P5** | **+13.4%** | **t(1757) = -139.8, *p* < .001*** |
| **P6** | **-3.8%** | **t(1524) = 58.9, p < .001*** | **P6** | **+1.8%** | **t(1757) = -18.7, *p* < .001*** |
| **P7** | **-2.9%** | **t(1524) = 45.5, *p* < .001*** | **P7** | **-1.6%** | **t(1757) = 16.6, *p* < .001*** |
| *Left primary motor cortex activation* | | | *Pre-supplementary motor area activation* | | |
| P1 | +1.4% | t(1778) = -0.4, *p* = 1 | P1 | +0.4% | t(1764) = -0.6, *p* = .794 |
| **P2** | -4.7% | t(1778) = 1.5, *p* = .441 | **P2** | **-10.1%** | **t(1764) = 3.2, *p* = .005*** |
| P3 | 0% | t(1778) < 0.1, *p* = 1 | P3 | 0% | t(1764) < 0.1, *p* = 1 |
| P4 | 0% | t(1778) < 0.1, *p* = 1 | P4 | 0% | t(1764) < 0.1, *p* = 1 |
| **P5** | -1.1% | t(1778) = -0.2, *p* = 1 | **P5** | **+5.8%** | **t(1764) = -3.6, *p* = .001*** |
| **P6** | **-27.9%** | **t(1778) = 8.7, *p* < .001*** | **P6** | **-19.1%** | **t(1764) = 5.9, *p* < .001*** |
| P7 | +0.7% | t(1778) = -0.3, *p* = 1 | P7 | -1.9% | t(1764) = 0.7, *p* = .844 |
| *Right insula activation* | | | *Left auditory cortex activation* | | |
| P1 | -0.04% | t(1785) = 0.2, *p* = 1 | P1 | +1.8% | t(1764) = -0.8, *p* = .740 |
| **P2** | **-10.5%** | **t(1785) = 4.9, *p* < .001*** | P2 | -1.8% | t(1764) = 0.6, *p* = .742 |
| P3 | 0% | t(1785) < 0.1, *p* = 1 | P3 | 0% | t(1764) < 0.1, *p* = 1 |
| P4 | 0% | t(1785) < 0.1, *p* = 1 | P4 | 0% | t(1764) < 0.1, *p* = 1 |
| P5 | +1.1% | t(1785) = -0.5, *p* = 1 | **P5** | **+6.9%** | **t(1764) = -3.5, *p* = .002*** |
| **P6** | **-12.7%** | **t(1785) = 5.8, *p* < .001*** | **P6** | **-10.0%** | **t(1764) = 4.2, *p* < .001*** |
| **P7** | **+7.5%** | **t(1785) = -3.5, *p* = .001*** | **P7** | **-6.1%** | **t(1764) = 2.8, *p* = .009*** |
| *Total file size* | | |  |  |  |
| **P1** | **-9.9%** | **t(1757) = 36.7, *p* < .001*** |  |  |  |
| **P2** | **-16.6%** | **t(1757) = 61.6, *p* < .001*** |  |  |  |
| **P3** | **+4.0%** | **t(1757) = -14.8, *p* < .001*** |  |  |  |
| **P4** | **+17.0%** | **t(1757) = -62.8, *p* < .001*** |  |  |  |
| **P5** | **+31.5%** | **t(1757) = -116.3, *p* < .001*** |  |  |  |
| **P6** | **+7.7%** | **t(1757) = -28.6, *p* < .001*** |  |  |  |
| **P7** | **+190.7%** | **t(1757) = -705.5, *p* < .001*** |  |  |  |

Note: * reflects significant results at *p* < .05. Significant results are also in bold. N = 257.

# Visualising variability and specificity

| 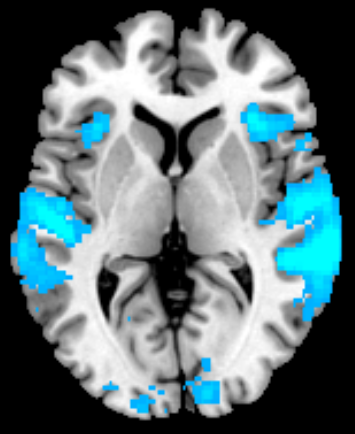 | 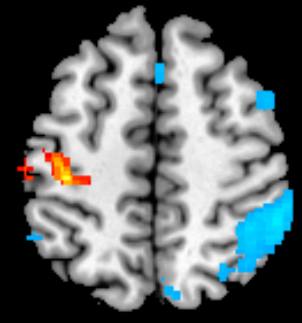 | 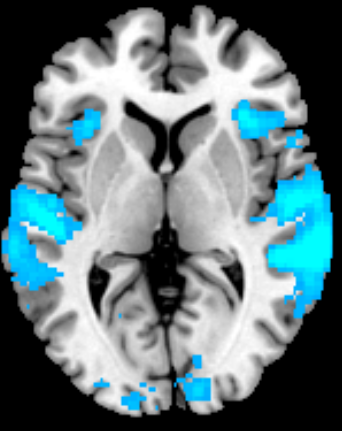 | 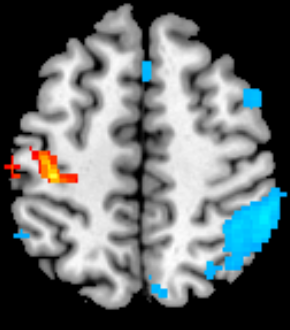 |
| --- | --- | --- | --- |
| Pipeline 8 – Increased parallelisation (supplementary) | | Pipeline 9 – Removed parallelisation (supplementary) | |

## *Supplementary Figure 7. Activation count maps for supplementary fMRIPrep pipelines modulating parallelisation. Values reflect the percentage of participants within the sample (N = 257) showing significant individual-level activation in a given voxel for both the ‘go > successful stop’ (motor; hot colours) and ‘successful stop > go’ (response inhibition; cool colours) contrasts. Slices presented are at MNI coordinates Z= 4, and Z = 52*

| 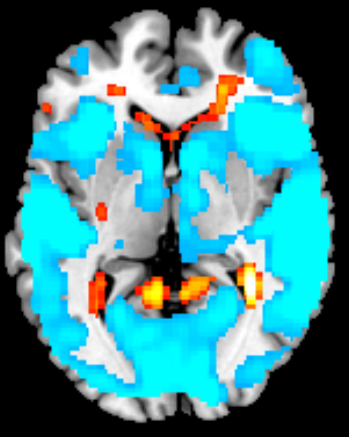 | 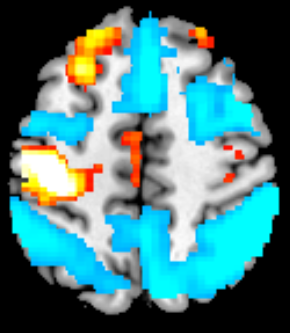 | | 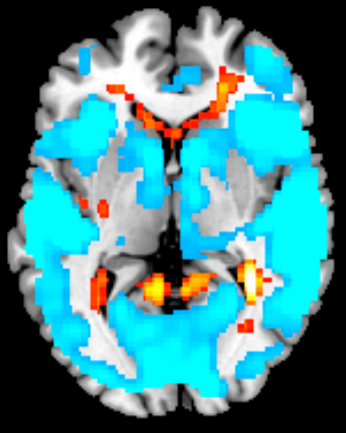 | 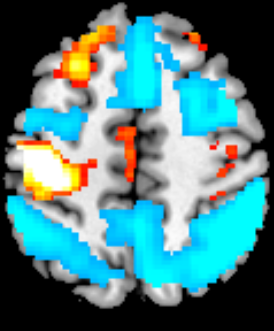 | 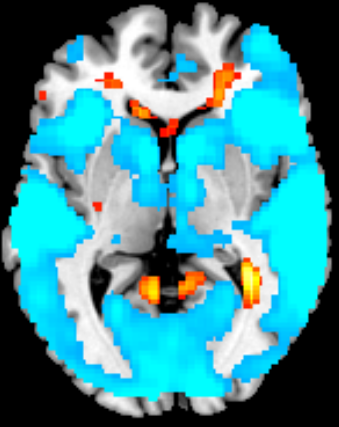 | | 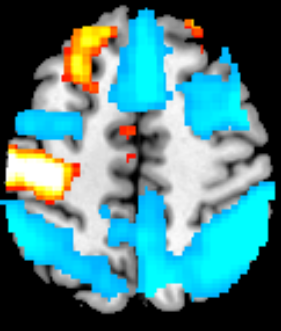 |  |
| --- | --- | --- | --- | --- | --- | --- | --- | --- |
| Pipeline 0 – Baseline | | | Pipeline 1 – No FreeSurfer surface reconstruction | | Pipeline 2 – ‘Sloppy’ registration | | |  |
| 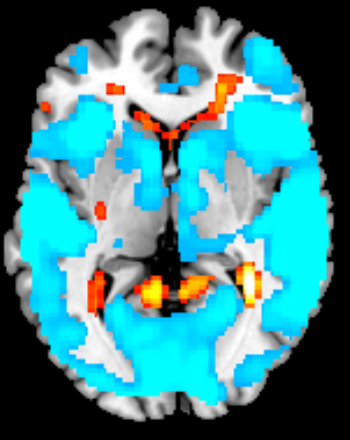 | 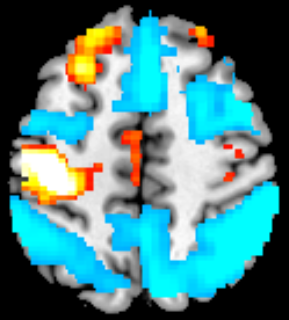 | | 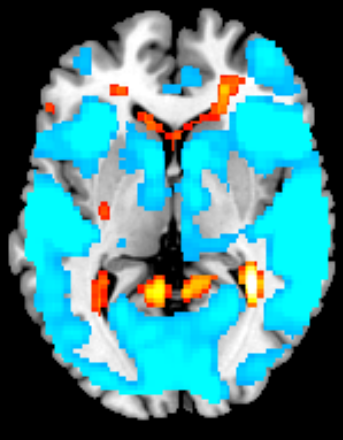 | 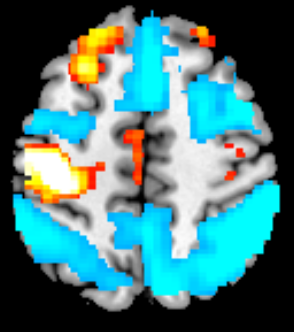 | 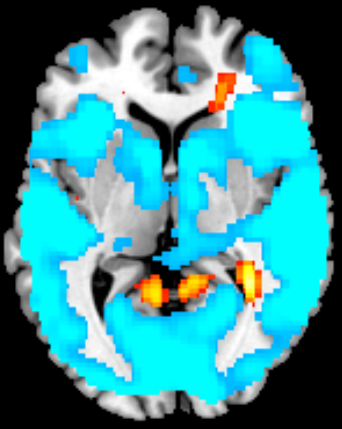 | | 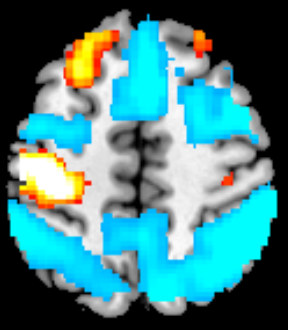 |  |
| Pipeline 3 – Low memory | | | Pipeline 4 – Surface output space added | | Pipeline 5 – ICA-AROMA | | |  |
| 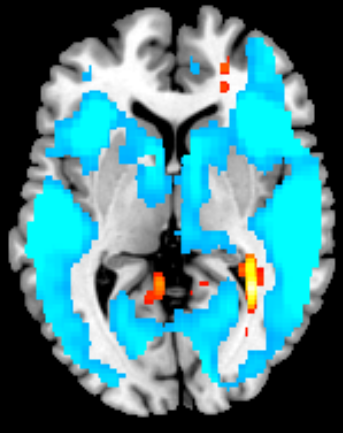 | | 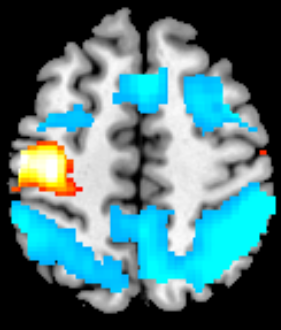 | | 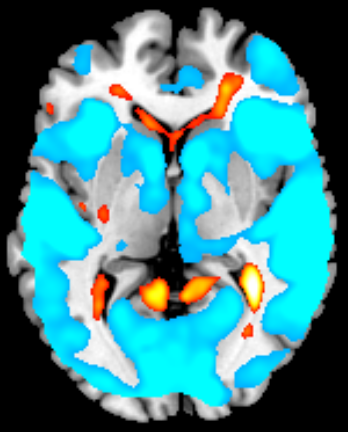 | | 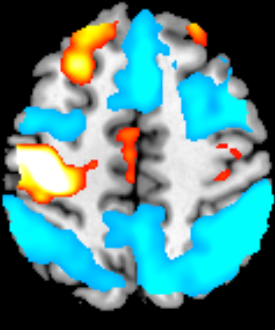 | |  |
| Pipeline 6 – Fieldmap-free distortion correction | | | | Pipeline 7 – Increased output space spatial resolution | | | | Pipeline 6 – Increased output space spatial resolution |
| 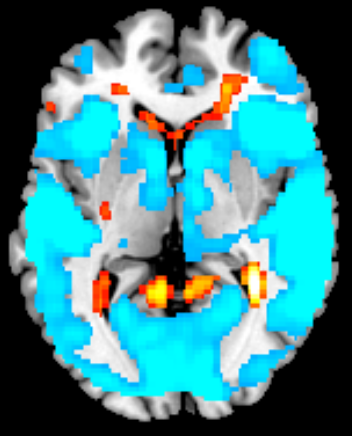 | | 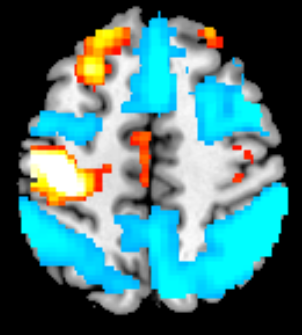 | | 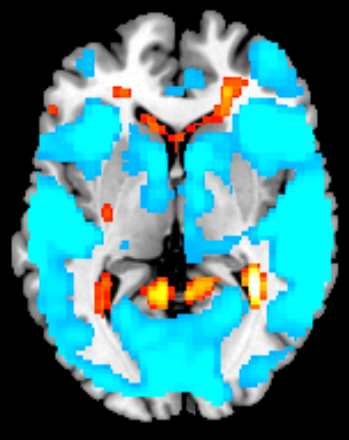 | | 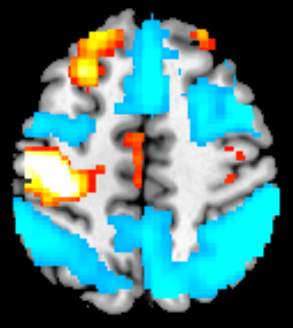 | |  |
| Pipeline 8 – Increased parallelisation (supplementary) | | | | Pipeline 9 – Removed parallelisation (supplementary) | | | |  |

## *Supplementary Figure 8. Thresholded group level analysis of activation associated with the contrast of ‘go > successful stop’ (hot colours) and ‘successful stop > go’ (cool colours). Analysis run in FSL FEAT, with a threshold of Z = 3.1, p = .05. Slices presented are at MNI coordinates Z= 4, and Z = 52. ICA = independent components analysis, AROMA = automatic removal of motion artefacts*

## Supplementary Table 6. Number of voxels in ‘go’ and ‘stop’ activation count maps (thresholded at 5%) and group-level analysis maps (thresholded at Z > 3.1) by pipeline

| **Pipeline** | **Number of voxels in thresholded map (percent change from baseline P0)** | | | |
| --- | --- | --- | --- | --- |
|  | **Activation count** | | **Group level activation** | |
|  | **‘Go’** | **‘Stop’** | **‘Go’** | **‘Stop’** |
| P0 | 634 | 11307 | 14315 | 59183 |
| P1 | 615 (-3.0%) | 10932 (-3.3%) | 13631 (-4.8%) | 55954 (-5.5%) |
| P2 | 628 (-0.9%) | 11653 (+3.1%) | 12648 (-11.6%) | 62154 (+5.0%) |
| P5 | 946 (+49.2%) | 17855 (+57.9%) | 11189 (-21.8%) | 67700 (+14.4%) |
| P6 | 454 (-28.4%) | 10936 (-3.3%) | 8076 (-43.6%) | 45331 (-23.4%) |
| P7* | 670 (+5.7%) | 11500 (+1.7%) | 14570 (+1.8%) | 59558 (+ 0.6%) |
| P8 | 637 (+0.5%) | 11301 (-0.1%) | 14282 (-0.2%) | 58987 (-0.3%) |

Note: Pipelines 3, 4, and 9 produced identical output to Pipeline 0, and as such are not included here. *Values for P7 are adjusted for the fact that images in the increased output resolution space have 8 times the number of voxels, overall. Corresponding values have therefore been divided by 8 here for the purposes of comparison.

| 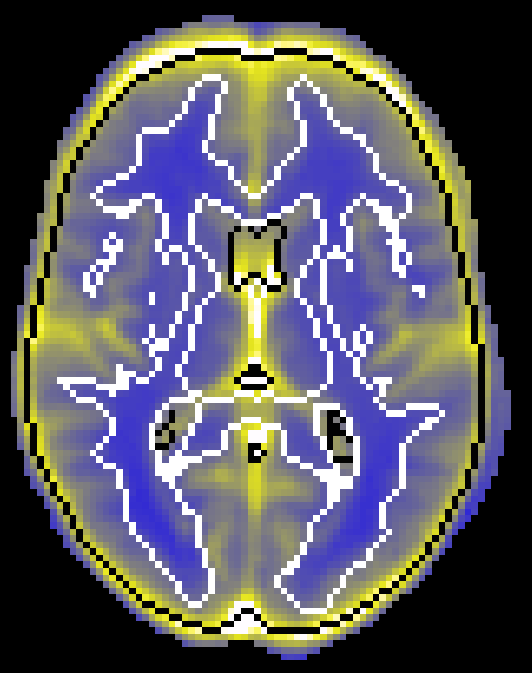 | 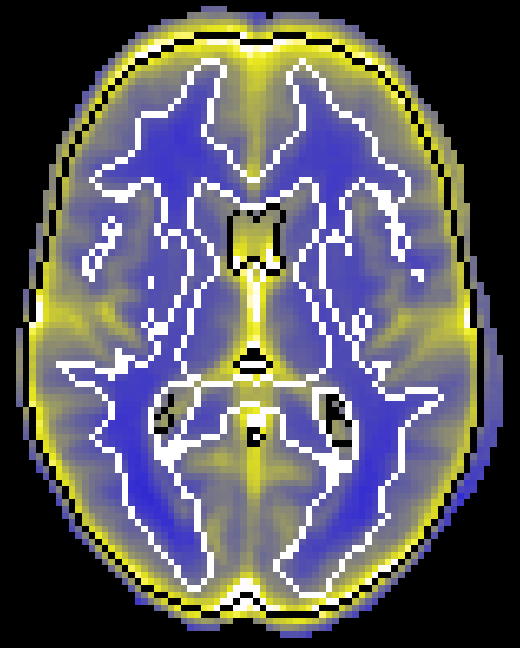 | 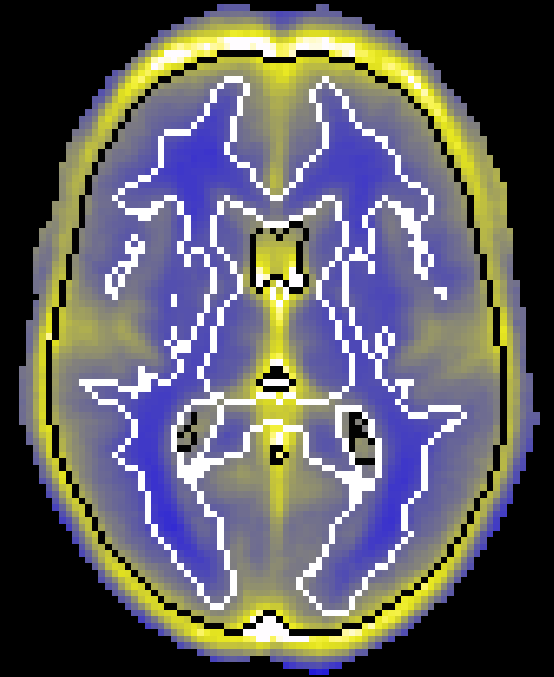 | 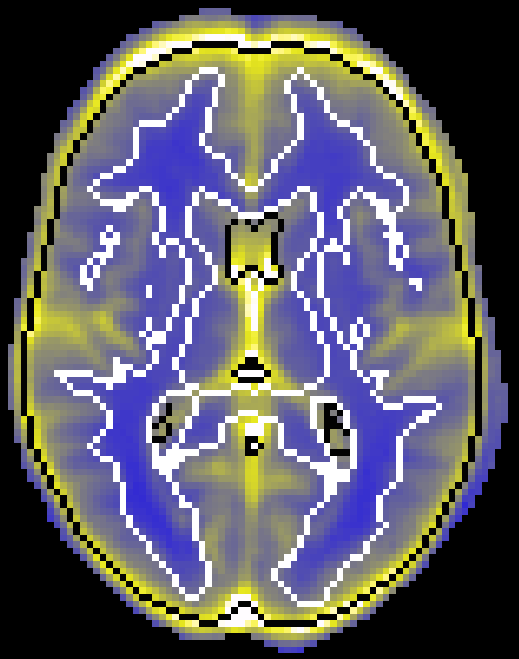 |
| --- | --- | --- | --- |
| Pipeline 0 – Baseline | Pipeline 1 – No FreeSurfer surface reconstruction | Pipeline 2 – ‘Sloppy’ registration | Pipeline 3 – Low memory |
| 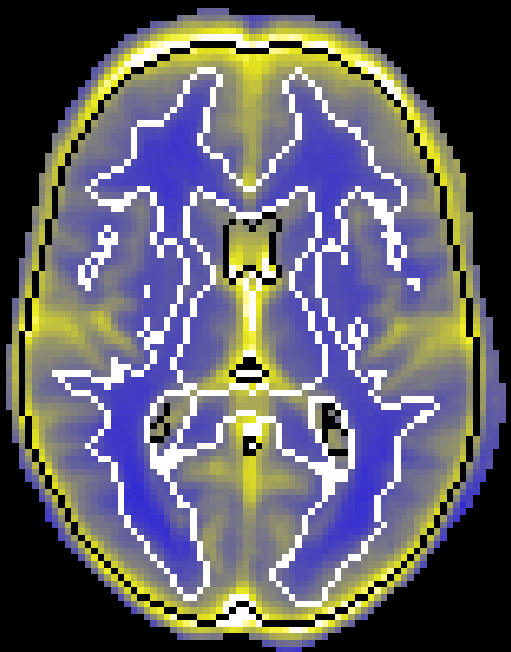 | N/A | 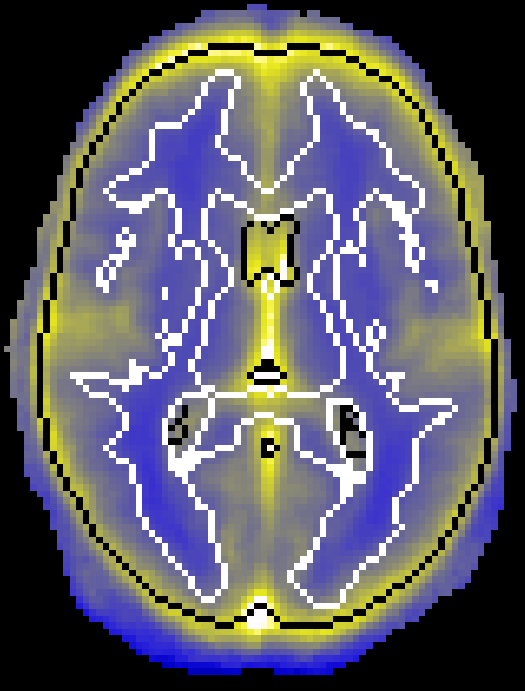 | 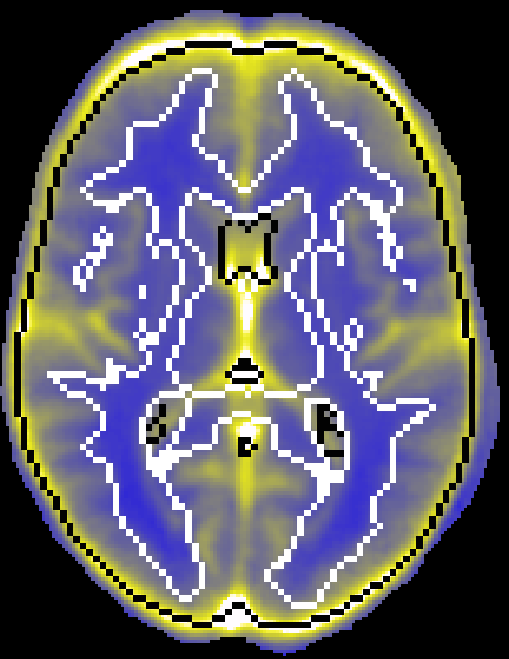 |
| Pipeline 4 – Surface output space added | Pipeline 5 – ICA-AROMA | Pipeline 6 – Fieldmap-free distortion correction | Pipeline 7 – Increased output space spatial resolution |
| 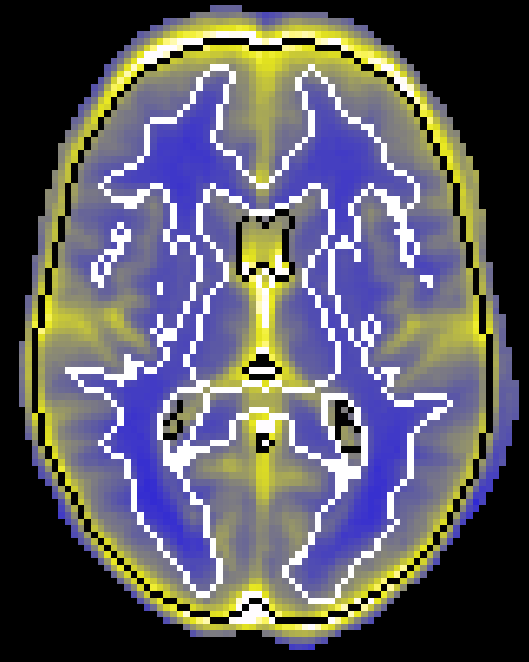 | | 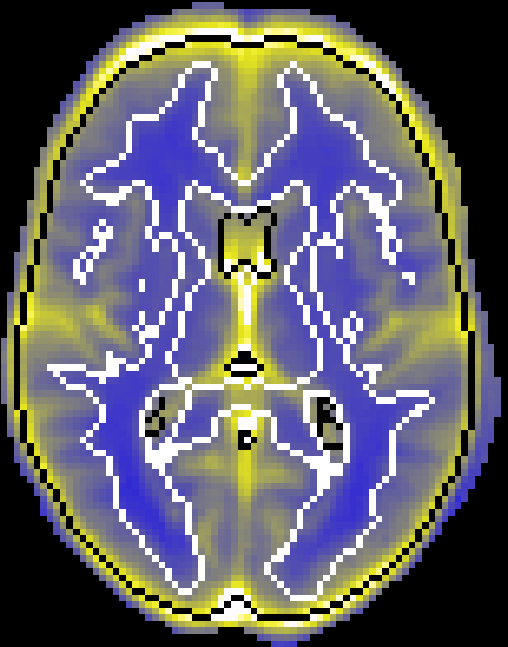 | |
| Pipeline 8 – Increased parallelisation (supplementary) | | Pipeline 9 – Removed parallelisation (supplementary) | |

## *Supplementary Figure 9. Timeseries standard deviation maps for each fMRIPrep pipeline. Values reflect the standard deviation of timeseries values within each voxel across the sample (N = 257). Lighter voxels reflect higher variability. Slices presented are at MNI coordinates Z = 10. Visualised using FSLEYES. The outline of the Brain in MNI space (black) as well as of white matter (white) are as defined by the Harvard-Oxford Subcortical Structural Atlas. It was not possible to produce a representative timeseries standard deviation map for pipeline 5, given that we did not have access to pre-smoothed maps for this pipeline. ICA = independent components analysis, AROMA = automatic removal of motion artefacts*

| 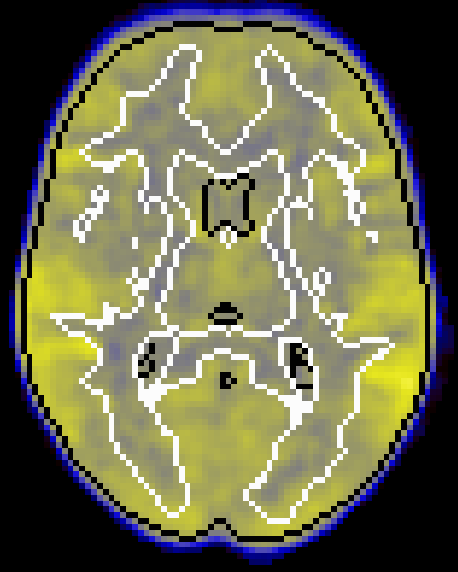 | 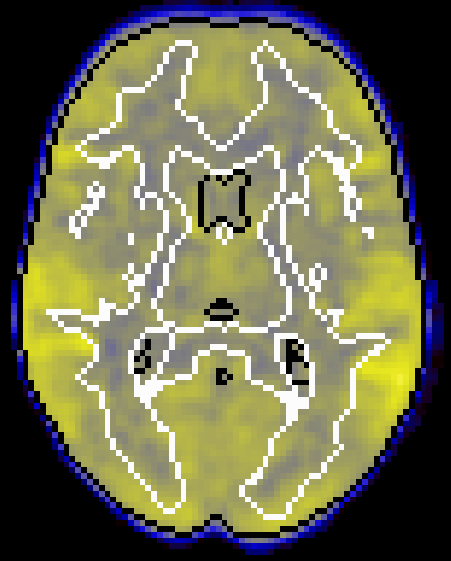 | 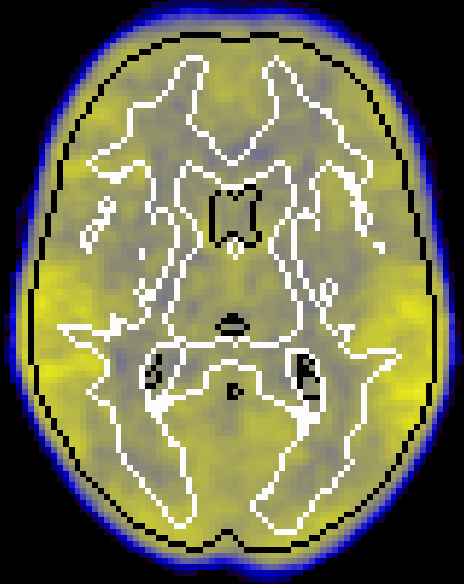 | 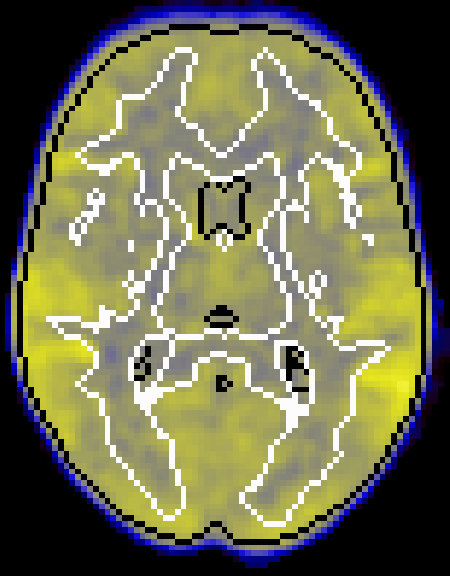 |
| --- | --- | --- | --- |
| Pipeline 0 – Baseline | Pipeline 1 – No FreeSurfer surface reconstruction | Pipeline 2 – ‘Sloppy’ registration | Pipeline 3 – Low memory |
| 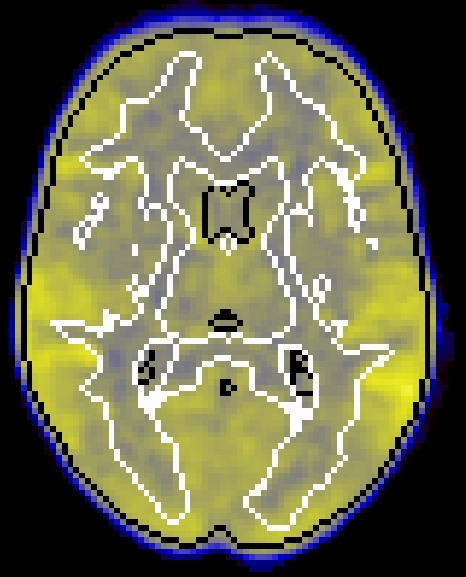 | 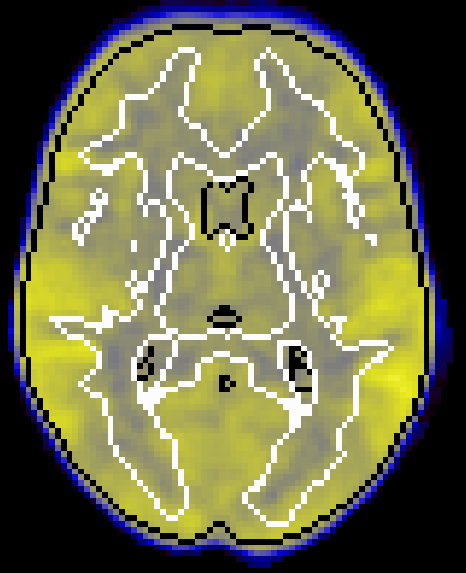 | 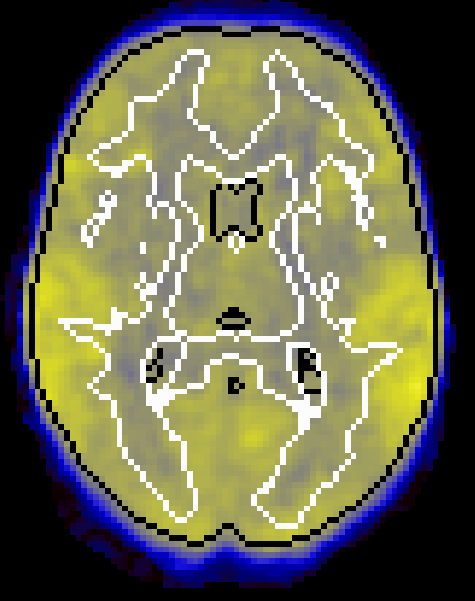 | 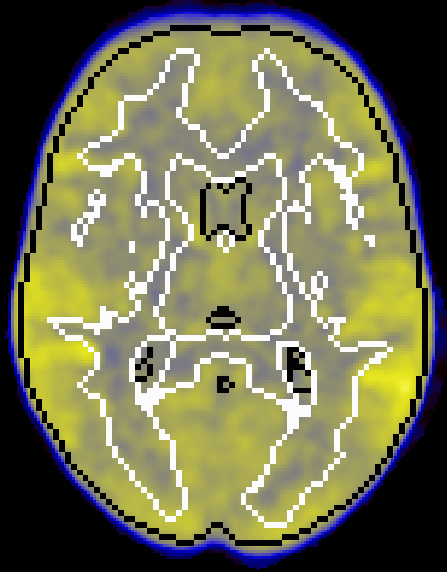 |
| Pipeline 4 – Surface output space added | Pipeline 5 – ICA-AROMA | Pipeline 6 – Fieldmap-free distortion correction | Pipeline 7 – Increased output space spatial resolution |
| 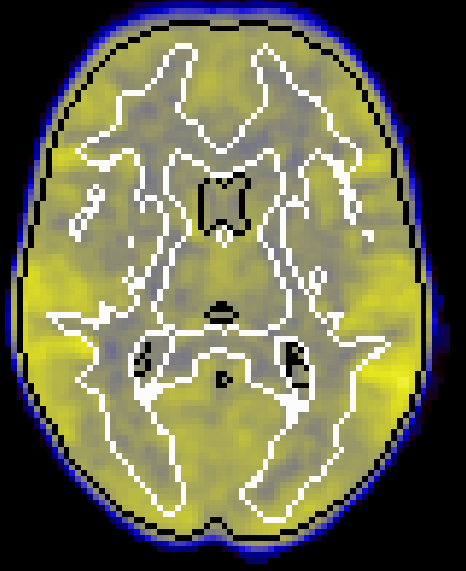 | | 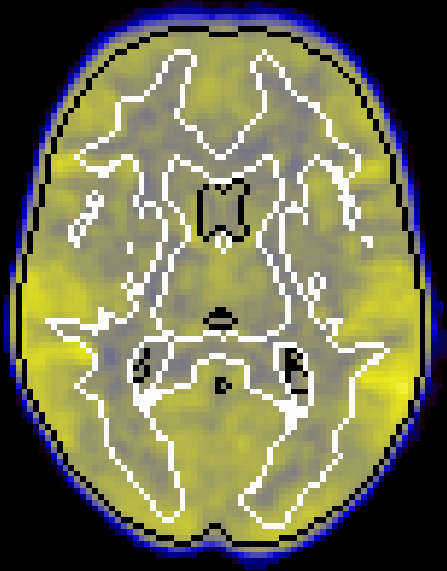 | |
| Pipeline 8 – Increased parallelisation (supplementary) | | Pipeline 9 – Removed parallelisation (supplementary) | |

## *Supplementary Figure 10. Activation standard deviation maps reflecting the standard deviation of z-statistics in each voxel, across the sample (N = 257), following first-level statistical analysis in FSL FEAT. Maps are derived from unthresholded files reflecting the contrast of go > successful stop. Lighter voxels reflect higher variability. Slices presented are at MNI coordinates Z = 10. Visualised using FSLEYES. The outline of the Brain in MNI space (black) as well as of white matter (white) are as defined by the Harvard-Oxford Subcortical Structural Atlas. ICA = independent components analysis, AROMA = automatic removal of motion artefacts*

## Supplementary Table 7. Mean standard deviation values of all voxels in each pipeline’s timseries standard deviation map and activation standard deviation map

|  | **Mean SD value (percent change from P0)** | |
| --- | --- | --- |
| **Pipeline** | **Timeseries SD map** | **Activation SD map** |
| P0 | 14.92 | 0.948 |
| P1 | 14.77 (-0.98%) | 0.942 (-0.6%) |
| P2 | 14.96 (+0.31%) | 0.946 (-0.2%) |
| P3 | 14.92 (+0%) | 0.948 (+/- 0%) |
| P4 | 14.92 (+0%) | 0.948 (+/- 0%) |
| P5 | N/A | 0.979 (+3.3%) |
| P6 | 14.44 (-3.23%) | 0.896 (-5.5%) |
| P7 | 14.92 (+0.04%) | 0.951 (+0.3%) |
| P8 | 14.92 (-0.01%) | 0.947 (-0.1%) |
| P9 | 14.92 (+0%) | 0.948 (+/- 0%) |

Note: Increases or decreases in average SD does not necessarily reflect lower or higher data quality. SD = standard deviation.

# References

Abraham, A., Pedregosa, F., Eickenberg, M., Gervais, P., Mueller, A., Kossaifi, J., Gramfort, A., Thirion, B., & Varoquaux, G. (2014). Machine learning for neuroimaging with scikit-learn. *Frontiers in Neuroinformatics, 8*. <https://doi.org/10.3389/fninf.2014.00014>

Avants, B. B., Epstein, C. L., Grossman, M., & Gee, J. C. (2008). Symmetric diffeomorphic image registration with cross-correlation: Evaluating automated labeling of elderly and neurodegenerative brain. *Medical Image Analysis, 12*(1), 26–41. <https://doi.org/10.1016/j.media.2007.06.004>

Behzadi, Y., Restom, K., Liau, J., & Liu, T. T. (2007). A component based noise correction method (CompCor) for BOLD and perfusion based fMRI. *NeuroImage, 37*(1), 90–101. <https://doi.org/10.1016/j.neuroimage.2007.04.042>

Country Specific Electricity Grid Greenhouse Gas Emission Factors [Internet]. carbonfootprint.com. 2022. Available from: <https://www.carbonfootprint.com/international_electricity_factors.html>

Dale, A. M., Fischl, B., & Sereno, M. I. (1999). Cortical surface-based analysis: I. Segmentation and surface reconstruction. *NeuroImage, 9*(2), 179–194. <https://doi.org/10.1006/nimg.1998.0395>

Davis, J., Bizo, D., Lawrence, A., Rogers, O., Smolaks, M., Simon, L., & Donnellan, D. (2022). *Uptime Institute Global Data Center Survey Results 2022*. <https://uptimeinstitute.com/resources/research-and-reports/uptime-institute-global-data-center-survey-results-2022>

Esteban, O., Blair, R., Markiewicz, C. J., Berleant, S. L., Moodie, C., Ma, F., Isik, A. I., et al. (2018). fMRIPrep 22.1.1 [*Software*]. <https://doi.org/10.5281/zenodo.852659>

Esteban, O., Markiewicz, C. J., Blair, R. W., Moodie, C. A., Isik, A. I., Erramuzpe, A., Kent, J. D., Goncalves, M., DuPre, E., Snyder, M., Oya, H., Ghosh, S. S., Wright, J., Durnez, J., Poldrack, R. A., & Gorgolewski, K. J. (2019). fMRIPrep: a robust preprocessing pipeline for functional MRI. *Nature Methods, 16*, 111-116. <https://doi.org/10.1038/s41592-018-0235-4>

Evans, A. C., Janke, A. L., Collins, D. L., & Baillet, S. (2012). Brain templates and atlases. *NeuroImage, 62*(2), 911–922. <https://doi.org/10.1016/j.neuroimage.2012.01.024>

Fonov, V. S., Evans, A. C., McKinstry, R. C., Almli, C. R., & Collins, D. L. (2009). Unbiased nonlinear average age-appropriate brain templates from birth to adulthood. *NeuroImage, 47*(Suppl 1), S102. <https://doi.org/10.1016/S1053-8119(09)70884-5>

Gorgolewski, K., Burns, C. D., Madison, C., Clark, D., Halchenko, Y. O., Waskom, M. L., & Ghosh, S. (2011). Nipype: A flexible, lightweight and extensible neuroimaging data processing framework in Python. *Frontiers in Neuroinformatics, 5*, 13. <https://doi.org/10.3389/fninf.2011.00013>

Gorgolewski, K. J., Esteban, O., Markiewicz, C. J., Ziegler, E., Ellis, D. G., Notter, M. P., Jarecka, D., et al. (2018). Nipype. *Software*. <https://doi.org/10.5281/zenodo.596855>

Goyal-Kamal, Feld, B., Schmidt, V., Goyal, K., Zhao, F., Joshi, A., Luccioni, S., Laskaris, N., Connell, L., Wang, Z., Catovic, A., Blank, D., Stęchły, M., J. P. W., Amine, S., & kraktus. (2021). CodeCarbon: Estimate and track carbon emissions from machine learning computing. *Zenodo*. Available at: <https://doi.org/10.5281/zenodo.4699491>

Greve, D. N., & Fischl, B. (2009). Accurate and robust brain image alignment using boundary-based registration. *NeuroImage, 48*(1), 63–72. <https://doi.org/10.1016/j.neuroimage.2009.06.060>

Huntenburg, J. M. (2014). *Evaluating nonlinear coregistration of BOLD EPI and T1w images* [Master's thesis, Freie Universität]. <http://hdl.handle.net/11858/00-001M-0000-002B-1CB5-A>

Jenkinson, M., Bannister, P., Brady, M., & Smith, S. (2002). Improved optimization for the robust and accurate linear registration and motion correction of brain images. *NeuroImage, 17*(2), 825–841. <https://doi.org/10.1006/nimg.2002.1132>

Klein, A., Ghosh, S. S., Bao, F. S., Giard, J., Häme, Y., Stavsky, E., Lee, N., et al. (2017). Mindboggling morphometry of human brains. *PLOS Computational Biology, 13*(2), e1005350. <https://doi.org/10.1371/journal.pcbi.1005350>

Lanczos, C. (1964). Evaluation of noisy data. *Journal of the Society for Industrial and* *Applied Mathematics Series B Numerical Analysis, 1*(1), 76–85. <https://doi.org/10.1137/0701007>

Patriat, R., Reynolds, R. C., & Birn, R. M. (2017). An improved model of motion-related signal changes in fMRI. *NeuroImage, 144*(Part A), 74–82. <https://doi.org/10.1016/j.neuroimage.2016.08.051>

Power, J. D., Mitra, A., Laumann, T. O., Snyder, A. Z., Schlaggar, B. L., & Petersen, S. E. (2014). Methods to detect, characterize, and remove motion artifact in resting state fMRI. *NeuroImage, 84*(Supplement C), 320–341. <https://doi.org/10.1016/j.neuroimage.2013.08.048>

Pruim, R. H. R., Mennes, M., van Rooij, D., Llera, A., Buitelaar, J. K., & Beckmann, C. F. (2015). ICA-AROMA: A robust ICA-based strategy for removing motion artifacts from fMRI data. *NeuroImage, 112*(Supplement C), 267–277. <https://doi.org/10.1016/j.neuroimage.2015.02.064>

Satterthwaite, T. D., Elliott, M. A., Gerraty, R. T., Ruparel, K., Loughead, J., Calkins, M. E., Eickhoff, S. B., Hakonarson, H., Gur, R. C., Gur, R. E., & Bassett, D. S. (2013). An improved framework for confound regression and filtering for control of motion artifact in the preprocessing of resting-state functional connectivity data. *NeuroImage, 64*(1), 240-256. <https://doi.org/10.1016/j.neuroimage.2012.08.052>

Treiber, J. M., White, N. S., Steed, T. C., Bartsch, H., Holland, D., Farid, N., McDonald, C. R., Carter, B. S., Dale A. M., & Chen, C. C. (2016). Characterization and correction of geometric distortions in 814 diffusion weighted images. *PLoS ONE, 11*(3), e0152472. <https://doi.org/10.1371/journal.pone.0152472>

Tustison, N. J., Avants, B. B., Cook, P. A., Zheng, Y., Egan, A., Yushkevich, P. A., & Gee, J. C. (2010). N4ITK: improved N3 bias correction. *IEEE Transactions on Medical Imaging, 29*(6), 1310-1320. <https://doi.org/10.1109/TMI.2010.2046908>

Wang, S., Peterson, D. J., Gatenby, J. C., Li, W., Grabowski, T. J., & Madhyastha, T. M. (2017). Evaluation of field map and nonlinear registration methods for correction of susceptibility artifacts in diffusion MRI. *Frontiers in Neuroinformatics, 11*. <https://doi.org/10.3389/fninf.2017.00017>

Zhang, Y., Brady, M., & Smith, S. (2001). Segmentation of brain MR images through a hidden Markov random field model and the expectation-maximization algorithm. *IEEE Transactions on Medical Imaging, 20*(1), 45-57. <https://doi.org/10.1109/42.906424>

1. PUE will vary based on the time of year at which it is measured. Estimated emissions for the computing conduct here may be considerably higher at other institutions, given that 1.28 is considerably lower than the PUE industry standard of 1.55, as reported for 2022 (Davis et al., 2022). [↑](#footnote-ref-1)
